# Flow Cytometry Illuminates Dental Stem Cells: a Systematic Review of Immunomodulatory and Regenerative Breakthroughs

**DOI:** 10.1007/s12015-025-10883-y

**Published:** 2025-04-25

**Authors:** Robert B. Stieger, Bledar Lilaj, Gernot P. Hönigl, Sophie Pock, Barbara Cvikl

**Affiliations:** Department of Conservative Dentistry, Sigmund Freud University, Vienna, Austria

**Keywords:** Dental stem cells, Flow cytometry, Regenerative medicine, Immunomodulation, Tissue regeneration

## Abstract

**Background:**

Dental stem cells hold significant potential in regenerative medicine due to their multipotency, accessibility, and immunomodulatory effects. Flow cytometry is a critical tool for analyzing these cells, particularly in identifying and characterizing immunomodulatory markers that enhance their clinical applications. This systematic review aims to answer the question: *"How does flow cytometry facilitate the identification and characterization of immunomodulatory markers in dental stem cells to enhance their application in regenerative medicine?"*.

**Methods:**

An exhaustive literature search was conducted in PubMed, retrieving 430 studies, of which 284 met inclusion criteria. Studies were selected based on the use of flow cytometry to analyze immunomodulatory markers in dental stem cells, focusing on methodologies, key findings, and challenges.

**Results:**

Of the 284 articles, 229 employed flow cytometry, with 115 reporting relevant results. Flow cytometry revealed important insights into the immunological interactions of various dental stem cells, including dental pulp stem cells, stem cells from human exfoliated deciduous teeth, periodontal ligament stem cells, and stem cells from the apical papilla, by identifying and characterizing immunomodulatory markers such as PD-L1, IDO, and TGF-β1.

**Conclusions:**

Flow cytometry is essential for advancing the understanding of dental stem cells' immunomodulatory properties. Standardization of methodologies is required to overcome technical challenges and enhance the clinical applications of dental stem cells in regenerative medicine and immunotherapy.

**Graphical Abstract:**

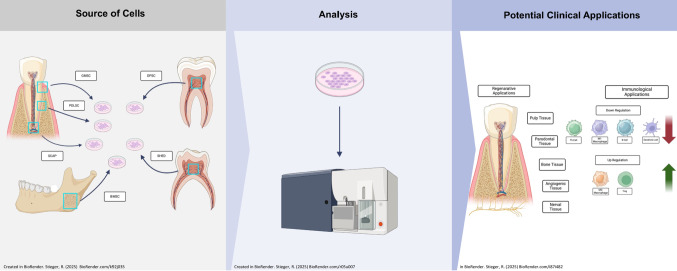

## Introduction

**Fig. 1:**
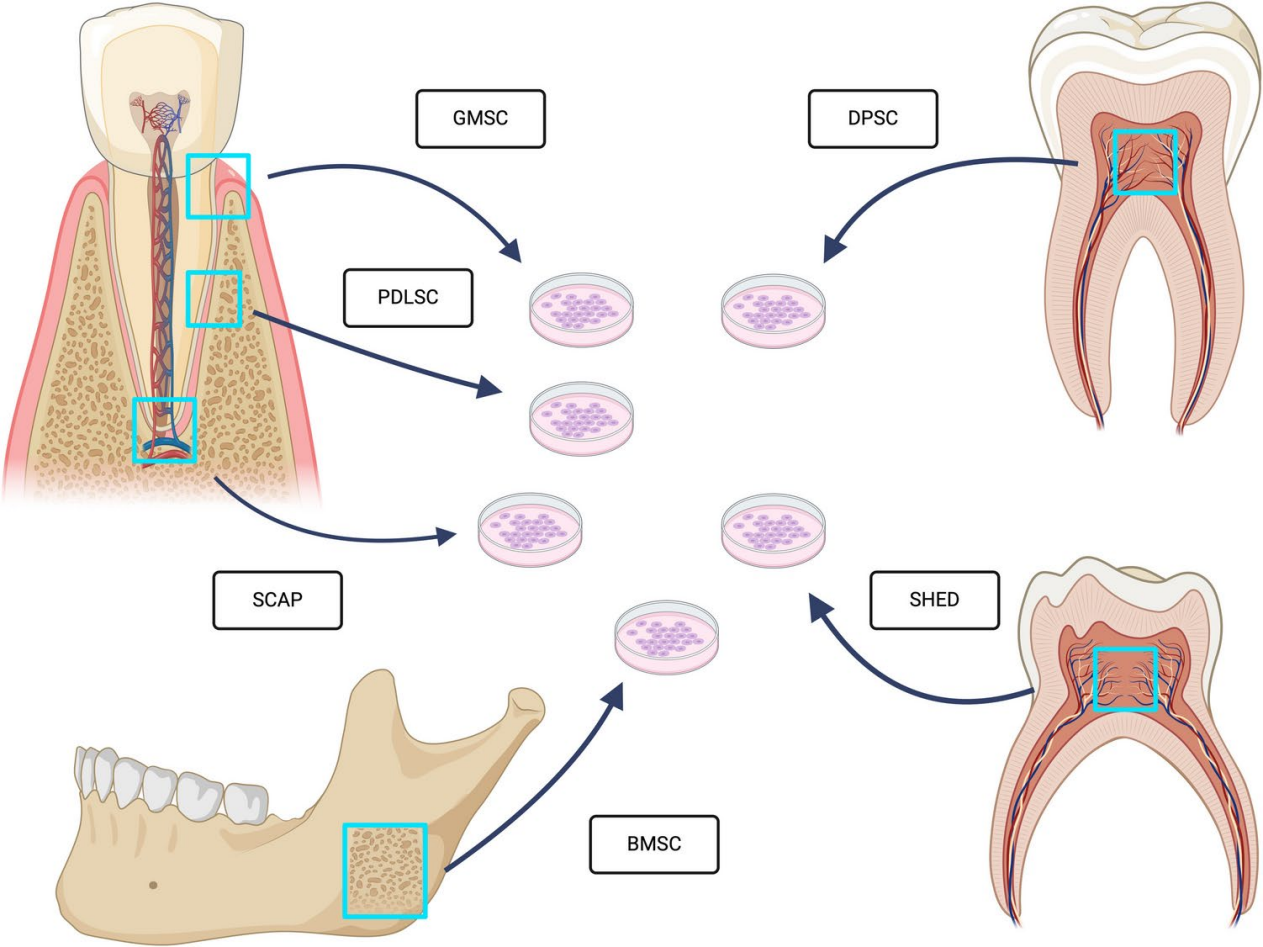
Shows the different sources of dental and craniofacial stem cells, including GMSC, PDLSC, SCAP, BMSC, DPSC, and SHED, highlighting their anatomical origins. These stem cells are cultured in vitro for potential applications in tissue regeneration Created in BioRender. Stieger, R. (2025) https://BioRender.com/k92j035

Dental stem cells have garnered significant attention in the field of regenerative medicine due to their remarkable properties, including multipotency, high proliferative capacity, and ease of accessibility. These cells are derived from various dental tissues such as dental pulp, periodontal ligament, exfoliated deciduous teeth, apical papilla, and gingival tissues. The primary types of dental stem cells include dental pulp stem cells (DPSCs), stem cells from human exfoliated deciduous teeth (SHED), periodontal ligament stem cells (PDLSCs), stem cells from the apical papilla (SCAP), gingival mesenchymal stem cells (GMSCs), and bone marrow mesenchymal stem cells (BMSCs) as shown in Fig. 1. Their unique characteristics have propelled research efforts aiming at harnessing their potential for regenerating dental tissues and beyond. However, despite extensive research on dental stem cells, a systematic evaluation of how flow cytometry contributes to their characterization and clinical potential remains lacking. This study aims to address this gap by critically reviewing the application of flow cytometry in dental stem cell research, focusing on methodologies, key findings, and the challenges that must be overcome for clinical translation.

### Background Dental Stem Cells

Dental stem cells are a subset of mesenchymal stem cells (MSCs) that originate from craniofacial neural crest cells during embryonic development. Isolated from postnatal dental tissues, these cells offer a less controversial and more ethically acceptable source compared to embryonic stem cells [13]. They exhibit several advantageous properties.

**Fig. 2:**
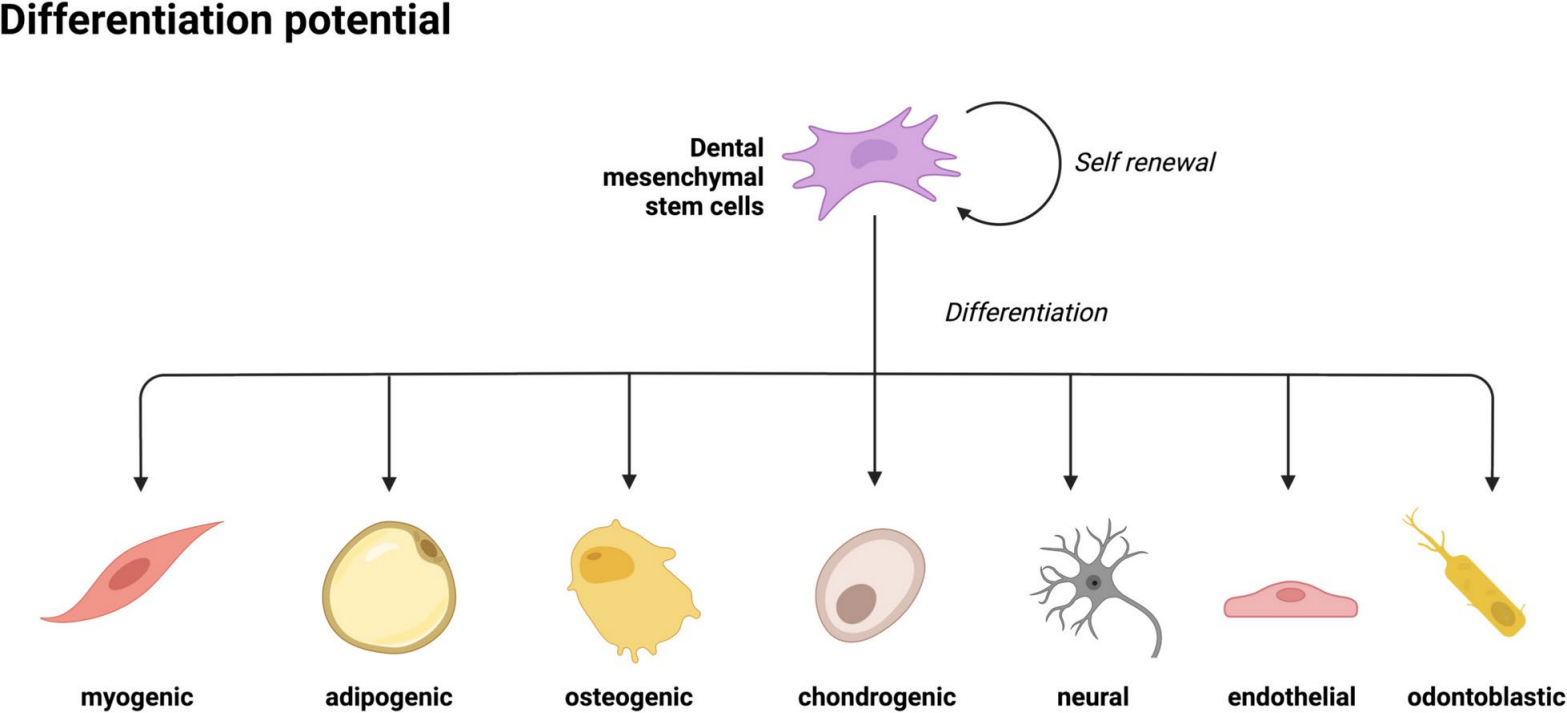
Shows the Differentiation potential of Mesenchmal stem cells (MSC) Created in BioRender. Stieger, R. (2025) https://BioRender.com/r77u840

Firstly, their multipotency sallows them to differentiate into a variety of cell types, including odontoblasts [42], osteoblasts [18], chondrocytes [65], adipocytes [29], neural cells [43], and endothelial cells [11] as shown in Fig. 2. This capacity is crucial for regenerating complex tissues and organs [52].

Secondly, they have a high proliferative capacity, enabling extensive *in vitro* expansion while maintaining their undifferentiated state, which is essential for generating sufficient cell numbers for therapeutic applications [50].

**Fig. 3:**
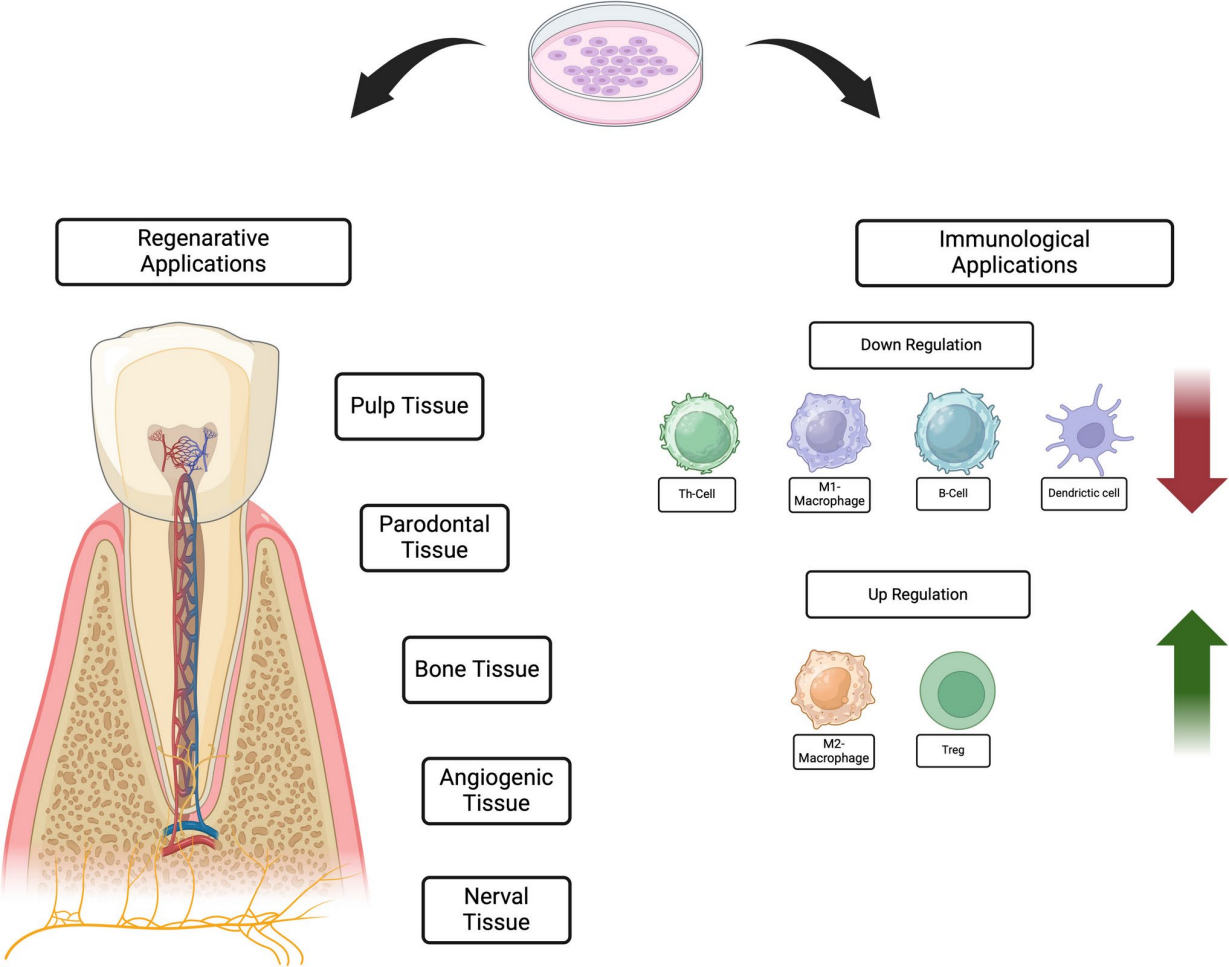
The figure illustrates the dual roles of dental stem cells in regenerative and immunological applications. On the regenerative side, dental stem cells contribute to the repair and regeneration of pulp, periodontal, bone, angiogenic, and neural tissues and their up- and downregulation [75] Created in BioRender. Stieger, R. (2025) https://BioRender.com/i87i482

Thirdly, their immunomodulatory effects enable them to interact with immune cells—including T cells, B cells, natural killer cells, and macrophages—which often leads to beneficial immunosuppression that reduces inflammation and promotes tissue regeneration [17, 47, 74, 75]. These properties, including their regenerative capacity and immunomodulatory effects, are summarized in Fig. 3.

Lastly, their ease of accessibility—as dental tissues like exfoliated deciduous teeth and extracted third molars are readily available—makes them an abundant and non-invasive source of stem cells with minimal ethical concerns [14].

The unique properties of dental stem cells have led to their exploration in various regenerative therapies. For instance, DPSCs have been investigated for regenerating dental pulp tissue, repairing bone defects, and treating neurodegenerative diseases [2, 14]. SHEDs have shown promise in promoting angiogenesis and neurogenesis, vital for tissue repair and regeneration [31]. PDLSCs are being studied for their potential to regenerate periodontal tissues, which could revolutionize periodontal disease treatment [49].

### Dental Stem Cells and their challenges

Despite their potential, several challenges hinder the clinical translation of dental stem cells. A primary challenge is the heterogeneity of stem cell populations derived from dental tissues, which can affect the consistency and efficacy of therapeutic outcomes [21, 34]. Variability in isolation and culture protocols across different studies leads to inconsistent results and difficulties in reproducing findings. Additionally, the limited understanding of the molecular mechanisms underlying the differentiation and immunomodulatory capabilities of dental stem cells impedes the optimization of their therapeutic use [9]. Regulatory and safety concerns, particularly regarding the risk of tumorigenicity and immune reactions, also pose significant hurdles that require rigorous testing before widespread clinical application.

### Flow Cytometry in Dental Stem Cell Research

Addressing these challenges necessitates comprehensive characterization and functional assessment of dental stem cells, which can be facilitated by advanced analytical techniques such as flow cytometry. Flow cytometry is a powerful tool that allows for rapid, quantitative, and multiparametric analysis of physical and chemical characteristics of cells in heterogeneous populations. In dental stem cell research, flow cytometry, especially fluorescence-activated cell sorting (FACS), plays a pivotal role in immunophenotyping, cell sorting, functional analysis, and studying cell–cell interactions.

By using established markers like CD73, CD90, and CD105 (positive markers) [27] and CD34 and CD45 (negative markers) [62], researchers can reliably identify MSCs from dental tissues, aligning with the criteria set by the International Society for Cellular Therapy (ISCT) [79]. Flow cytometry enables the analysis of heterogeneity within dental stem cell populations by quantifying the expression of various markers associated with stemness, differentiation potential, and immunomodulatory properties. It allows for the assessment of the differentiation status of stem cells into various lineages by staining for lineage-specific markers. Additionally, flow cytometry helps evaluate the expression of immunomodulatory molecules (e.g., HLA-G, PD-L1) [22, 65] and the secretion of cytokines involved in immune regulation [83] (e.g., IL- 10, TGF-β1) [8, 59]. This assessment is essential for understanding their role in immunomodulation and tissue regeneration [3, 84].

However, the application of flow cytometry in dental stem cell research is not without challenges. Technical variability due to differences in equipment, fluorochrome selection, antibody quality, and operator expertise can lead to inconsistent results. Preparing single-cell suspensions from dental tissues can be challenging because of the extracellular matrix components, which may affect cell viability and fluorescence staining. The choice of markers can influence the interpretation of results, highlighting the need for consensus on the most relevant markers for specific applications. Analyzing the complex data generated by flow cytometry requires sophisticated techniques. Variations in gating strategies and data interpretation can result in discrepancies between studies.

Given the potential of dental stem cells in regenerative medicine and the critical role of flow cytometry in their study, there is a pressing need for a systematic review that synthesizes current knowledge, identifies gaps and inconsistencies, promotes standardization, and facilitates clinical translation. This systematic review aims to consolidate and critically evaluate the existing literature on the application of flow cytometry in dental stem cell research, focusing on both immunological and regenerative applications.

### Rationale for the Systematic Review

Despite the significant potential of dental stem cells (DSCs) in regenerative medicine, a systematic evaluation of how flow cytometry contributes to their immunomodulatory characterization remains lacking. Understanding the immunomodulatory properties of DSCs is crucial, as these properties influence their therapeutic efficacy in tissue regeneration and immunotherapy. Flow cytometry, with its ability to analyze multiple parameters at the single-cell level, is a powerful tool for identifying and characterizing immunomodulatory markers on dental stem cells.

The primary objectives of this review are:

1. To examine the flow cytometry methodologies used in dental stem cell research, including sample preparation, antibody selection, staining protocols, and data analysis techniques, as described in the selected articles.
2. To evaluate how flow cytometry has contributed to understanding the immunomodulatory properties of dental stem cells, including interactions with immune cells and the expression of immunoregulatory molecules.
3. To identify the technical limitations and challenges associated with using flow cytometry in dental stem cells research, proposing solutions or recommendations where possible.
4. To discuss the implications of the findings for clinical practice, including potential applications in regenerative medicine and immunotherapy.

By systematically reviewing and synthesizing the literature, this study aims to enhance understanding of the current state of dental stem cell research using flow cytometry. It seeks to guide future research by identifying areas requiring further investigation and to support standardization efforts critical for advancing the field and facilitating regulatory approval for clinical applications. Additionally, this review intends to promote collaboration among researchers, clinicians, and industry partners by providing a common knowledge base. Ultimately, it contributes to the development of effective DSC-based therapies that can improve outcomes for patients with dental and systemic diseases.

## Material and Methods

This systematic review was conducted following the Preferred Reporting Items for Systematic Reviews and Meta-Analyses (PRISMA) guidelines [53].

Our primary research question was:"How does flow cytometry facilitate the identification and characterization of immunomodulatory markers in dental stem cells to enhance their application in regenerative medicine?”. The methodology was carefully designed to ensure comprehensive coverage and unbiased synthesis of literature focusing on the application of flow cytometry in analyzing immunomodulatory markers of dental stem cells.

### Search Strategy

An exhaustive search strategy was developed to identify relevant peer-reviewed studies. The search was conducted in PubMed using a combination of Medical Subject Headings (MeSH) terms and free-text keywords to maximize the retrieval of pertinent studies. The search string applied was designed as follows. Search Terms: Population Terms:"Dental pulp stem cells"OR"DPSCs"; Intervention Terms:"Flow cytometry"OR"Immunophenotyping"OR"Cell sorting"; Outcome Terms:"Immunomodulation"OR"Immunoregulatory markers"OR"Immune properties". This led to a combined search string:

*("Dental stem cells"OR"DPSCs"OR"PDLSCs"OR"SHED"OR"SCAP") AND ("Flow cytometry"OR"Immunophenotyping"OR"Cell sorting") AND ("Regeneration"OR"Differentiation"OR"Immunomodulation")*

**Fig. 4:**
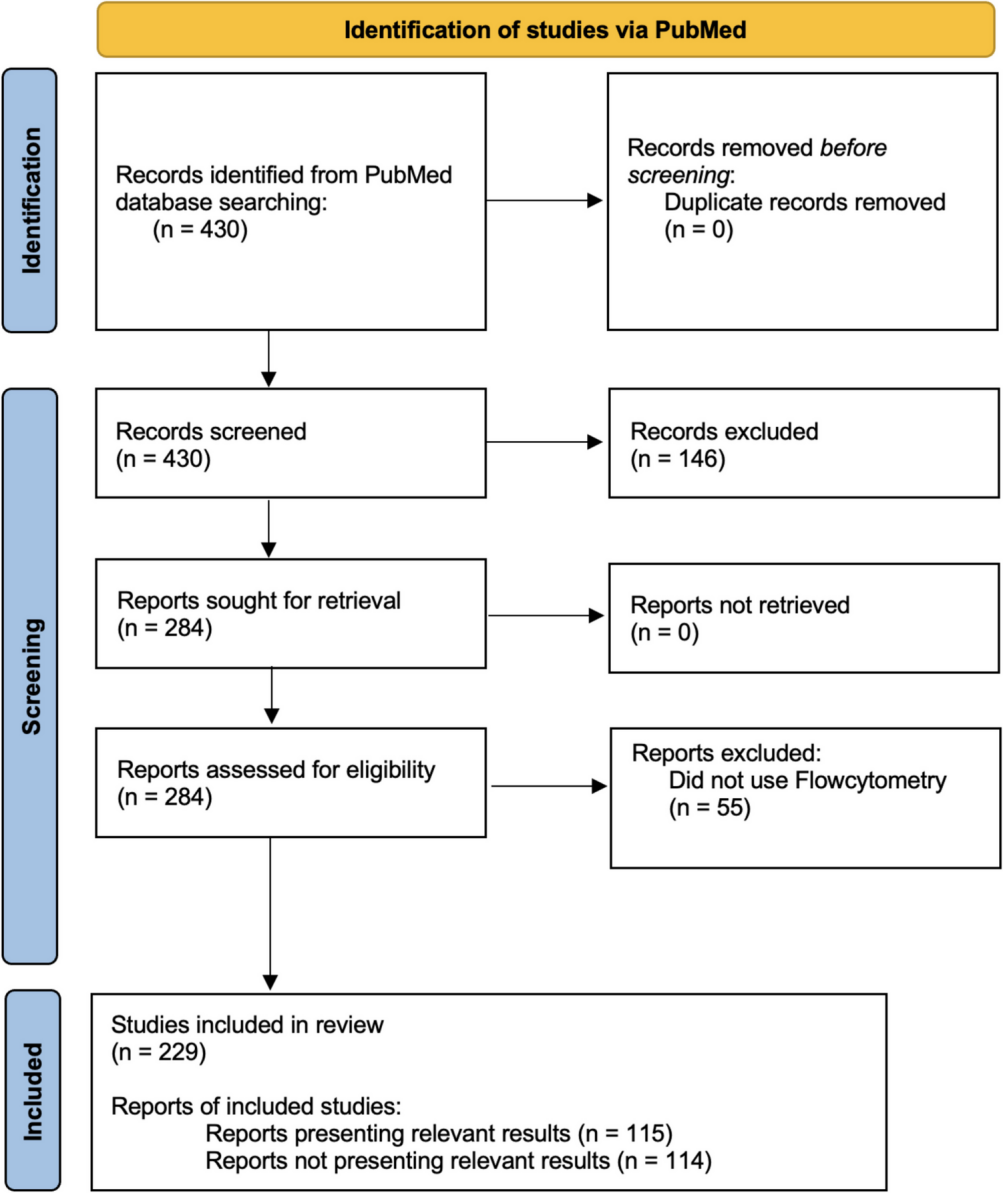
Shows the study selection and screening process according to the PRISMA guidelines [53]

The search was limited to original research articles published in English, published within 2010 and September 2024, to capture all relevant literature. The final search was conducted in September 2024 resulting in the retrieval of 430 articles as shown in Fig. 4.

### Study Selection Process

The study selection as shown in Fig. 3 involved a multi-phase approach, conducted independently by two reviewers. Initially, all 430 retrieved articles were screened based on their titles and abstracts to assess their relevance according to the inclusion criteria. Discrepancies between reviewers were resolved through discussion or consultation with a third reviewer if necessary. Following the initial screening, 284 articles were identified as potentially relevant.

These 284 articles were then further evaluated using a custom Python script designed to screen for specific methodological details related to flow cytometry applications in dental stem cell research. The script facilitated the extraction and organization of key data points, enhancing the efficiency of the screening process. To validate the results obtained from the Python script, both reviewers manually screened the 284 articles, ensuring the accuracy and reliability of the data extraction process. This combined approach allowed for a thorough and systematic evaluation of the literature, minimizing the risk of overlooking pertinent studies.

### Inclusion and Exclusion Criteria

We conducted a comprehensive analysis of original research articles utilizing flow cytometry to study dental stem cells. Our focus encompassed investigations on DPSCs, PDLSCs, SHED, SCAP, and DFPCs. Eligible studies explored key aspects such as immunophenotyping, differentiation potential, or immunomodulatory properties, with flow cytometry serving as the primary analytical method. Both *in vitro* and *in vivo* studies were included; they provided detailed essential flow cytometry methodologies, including marker selection, gating strategies, and antibody specifics.

To ensure the inclusion of studies presenting statistically meaningful results, we prioritized articles reporting significant findings, identified by the use of the term"significant"or p-values ≤ 0.05 in the abstract. Additionally, we extracted data on complementary methods used alongside flow cytometry, such as RT-PCR, qRT-PCR, alizarin red staining, and RNA sequencing, to provide a broader understanding of the experimental approaches. Exclusion criteria included review articles, meta-analyses, conference abstracts, and case reports. Studies focusing on non-dental stem cells or lacking sufficient details on flow cytometry methodology were also excluded.

### Definition of Relevant Results

In our analysis, the classification of"Relevant Results"was determined by a keyword-based approach focusing on the term"significant"and the presence of p-values equal to or less than 0.05.

### PICO Framework

The PICO (Population, Intervention, Comparison, Outcome) framework was employed to structure the research question and guide the systematic review process. The population comprised dental stem cells derived from human sources, specifically DPSCs, PDLSCs, SHED and SCAP. The intervention was the application of flow cytometry techniques, including immunophenotyping and cell sorting, to analyze the characteristics of these dental stem cells. There was no specific comparison group; however, comparisons were made within and across studies regarding different flow cytometry methodologies and markers used. The primary outcomes were the immunological and regenerative properties of dental stem cells as assessed by flow cytometry.

### Quality Assessment

The quality of the included studies was assessed using a modified version of the Strengthening the Reporting of Observational Studies in Epidemiology (STROBE) checklist, adapted for flow cytometry studies. The assessment criteria included flow cytometry methodology with clear descriptions of antibody selection and validation, staining procedures and, if reported, gating strategies, and documentation of instrument calibration and settings; reproducibility, with availability of sufficient methodological details to allow replication and use of standardized protocols or reference to established methods; controls, including the inclusion of appropriate positive and negative controls and the use of isotype controls and fluorescence-minus-one controls where applicable; and data reporting, with transparent presentation of results with appropriate statistical analysis, inclusion of representative flow cytometry plots and histograms, and discussion of potential sources of limitations.

### Data Extraction

A standardized data extraction form was developed to systematically collect relevant information from each included study. The data extraction process included general information such as title, authors, year of publication, and journal; methodology including stem cell type (DPSCs, PDLSCs, SHED, SCAP, or DFPCs), flow cytometry details (markers used, antibody specifics, staining procedures, gating strategies, instrument settings), and culture conditions (medium composition, supplements, culture duration); study design including *in vitro* or *in vivo* setup, sample size, and control groups; key findings such as immunophenotyping results, differentiation potential markers, and immunomodulatory findings; technical challenges including reported difficulties in flow cytometry protocols and data interpretation issues; and quality assessment based on methodological rigor and reporting standards. Data extraction was performed independently by two reviewers to ensure accuracy and reliability.

### Automated Data Extraction and Processing

The text extracted from the articles was meticulously cleaned and normalized to ensure consistency and accuracy. Advanced Natural Language Processing techniques were employed for named entity recognition, summarization, and keyword extraction. Key data—including identifiers, metadata, and methodological details—were systematically extracted and organized for analysis. Statistical analyses, such as trend and correlation analyses, were conducted, with results visualized using charts and heatmaps to facilitate interpretation. Data subsets were created to allow focused analysis on specific aspects of the research. The final data and reports were compiled and exported in user-friendly formats to enhance accessibility and usability. Further two independent reviewers manually confirmed all 284 papers and their correct selection process to ensure the best data quality.

### Data Synthesis

The extracted data were synthesized into key thematic categories. Immunophenotyping involved comparative analysis of surface marker expression profiles among different dental stem cell types, identification of common and unique markers expressed, and assessment of markers related to stemness and hematopoietic markers. Differentiation potential included evaluation of the expression of markers indicative of differentiation into osteogenic, odontogenic, adipogenic, and neurogenic lineages, and analysis of the effect of different culture conditions and stimuli on differentiation markers. Immunomodulatory properties encompassed examination of the interactions between dental stem cells and immune cells, analysis of cytokine and chemokine secretion profiles, and assessment of the capacity of dental stem cells to modulate immune responses. Comparative analyses were performed to identify patterns, consistencies, and discrepancies across studies, and the synthesis highlighted key findings, methodological variations, and areas requiring further investigation.

#### Risk of Bias

The potential for bias within and across the included studies was considered during the data synthesis process. Due to the large number of articles and practical constraints, a comprehensive risk of bias assessment was not conducted for all 284 papers. Instead, general factors that could introduce bias—such as selection methods, reporting transparency, and methodological rigor—were taken into account when interpreting the findings. This approach allowed for an awareness of possible biases like selection bias, performance bias, detection bias, and reporting bias, without performing a detailed assessment for each study. Acknowledging these potential sources of bias, the data synthesis aimed to provide a balanced overview of the literature, while the limitations section discusses how these biases might affect the interpretation of the results.

#### Limitations

A significant limitation identified in the reviewed studies was the focus on categorizing dental stem cells without further sorting them using fluorescence-activated cell sorting for subsequent culturing and functional assays. This omission limits the exploration of specific subpopulations within dental stem cells that may have distinct therapeutic potentials. Additionally, there was inconsistency in the culture media and conditions used across studies. Variations in medium composition, supplements such as growth factors and serum types, and culture duration can significantly affect cell behavior and experimental outcomes, complicating the comparability of results. These discrepancies underscore the need for standardized protocols in dental stem cell research to enhance reproducibility and facilitate the translation of findings into clinical applications.

As this study involved the analysis of data from previously published articles, formal ethical approval was not required. However, all efforts were made to respect intellectual property rights by appropriately citing original sources. The review process was conducted with integrity, transparency, and adherence to ethical guidelines for scholarly research.

#### Self-Bias Consideration

Given that all members of the research team are dental professionals, there is an inherent risk of self-bias influencing the study's design, data interpretation, and conclusions. Our clinical backgrounds and familiarity with dental stem cell research may inadvertently shape our perspectives, potentially prioritizing findings that align with our professional experiences. To mitigate this bias, we implemented several strategies: we adhered strictly to predefined inclusion and exclusion criteria; employed automated data extraction methods to enhance objectivity; and engaged independent reviewers outside of our immediate professional circle to validate our findings. By acknowledging our potential for self-bias and taking proactive measures to address it, we aimed to ensure the integrity, transparency, and credibility of our systematic review.

## Results

A total of 430 articles related to flow cytometry in dental stem cell research were initially identified from the PubMed database. After screening for relevance and removing duplicates, 284 articles remained for further analysis. An advanced script utilizing natural language processing models and statistical tools was employed to extract essential information from these articles, including study titles, journals, publication dates, methodologies, cell origins, relevant results, and unique identifiers.

From the 284 articles, 229 (80.6%) reported the use of flow cytometry (FC) in their methodologies. The remaining 55 articles were excluded from further analysis due to the absence of FC usage. Among the 229 FC-utilizing articles, 115 (50.2%) presented results deemed relevant. The other 114 articles did not provide relevant results and were thus excluded from specific outcome analyses. (Table 1).

**Table 1:** Summarizes the methodologies used in 229 FC-related studies

Methodology	Relevant Results (n = 115)	Non-Relevant Results (n = 114)	Total FC Articles (n = 229)
Flow Cytometry	115 (100.0%)	114 (100.0%)	229 (100.0%)
Western Blotting	45 (39.1%)	28 (24.6%)	73 (31.9%)
Osteogenic Differentiation Assays	36 (31.3%)	34 (29.8%)	70 (30.6%)
Alizarin Red Staining	23 (20.0%)	14 (12.3%)	37 (16.2%)
Immunofluorescence	17 (14.8%)	20 (17.5%)	37 (16.2%)
Cell Counting Assays	15 (13.0%)	—	15 (6.6%)
Reverse Transcription PCR (RT-PCR)	15 (13.0%)	—	15 (6.6%)
Quantitative PCR (qPCR)	12 (10.4%)	—	12 (5.2%)
Enzyme-Linked Immunosorbent Assay (ELISA)	10 (8.7%)	—	10 (4.4%)
Transfection Techniques	10 (8.7%)	—	10 (4.4%)

Within the subset of 115 relevant articles, all utilized flow cytometry as a primary method. Other frequently employed methodologies included western blotting in 45 studies, osteogenic differentiation assays in 36 studies, alizarin red staining in 23 studies, immunofluorescence in 17 studies, cell counting assays and reverse transcription PCR.

(RT-PCR) each in 15 studies, quantitative PCR (qPCR) in 12 studies, enzyme-linked immunosorbent assay (ELISA) in 10 studies, and transfection techniques in 10 studies.

The dental stem cell types investigated in these studies were diverse. DPSCs were the most commonly studied, appearing in 54 articles. PDLSCs were investigated in 26 studies, SHED in 20 studies, SCAP in 14 studies, MSCs in 10 studies, BMSCs in one study, ABMSCs in one study, and GMSCs in one study.

Regarding the focus of these studies, 48 articles (41.7%) explored immunological applications, while 69 articles (60%) investigated regenerative applications. Some studies addressed both applications simultaneously.

In contrast, the 114 articles that used FC but did not yield relevant results showed different trends. Among these, 47 studies focused on immunological applications, and 84 studies concentrated on regenerative applications. Common methodologies in this group included flow cytometry in all 114 studies, osteogenic differentiation in 34 studies, western blotting in 28 studies, immunofluorescence in 20 studies, and alizarin red staining in 14 studies. The stem cell types studied were DPSCs in 62 articles, PDLSCs in 30 articles, SHED in 16 articles, MSCs in 8 articles, SCAP in 3 articles, with other types appearing less frequently (Table 2).

**Table 2:** Stem cell types used in FC studies

Stem Cell Type	Relevant Results (n = 115)	Non-Relevant Results (n = 114)	Total FACS Articles (n = 229)
DPSCs	54 (47.0%)	62 (54.4%)	116 (50.7%)
PDLSCs	26 (22.6%)	30 (26.3%)	56 (24.5%)
SHED	20 (17.4%)	16 (14.0%)	36 (15.7%)
SCAP	14 (12.2%)	3 (2.6%)	17 (7.4%)
MSCs	10 (8.7%)	8 (7.0%)	18 (7.9%)
BMSCs	1 (0.9%)	—	1 (0.4%)
ABMSCs	1 (0.9%)	—	1 (0.4%)
GMSCs	1 (0.9%)	—	1 (0.4%)

Further analysis of the methodologies employed in the relevant studies reveals that flow cytometry was universally utilized across all categories, underscoring its fundamental role in dental stem cell research. In the regenerative applications category, western blotting was used in 29 out of 69 studies (42.0%), while osteogenic differentiation assays were employed in 21 studies (30.4%). Alizarin red staining appeared in 14 studies (20.3%), highlighting its significance in evaluating mineralization during osteogenesis. Immunofluorescence was utilized in 11 studies (15.9%), and cell counting was conducted in 10 studies (14.5%) (Table 3).

**Table 3:** Summarizes the methodologies used in FC-related studies with regenerative applications, immunological applications and with both regenerative and immunological applications

Method	Regenerative Applications (n = 69)	Immunological Applications (n = 48)	Both Applications (n = 31)
Flow Cytometry	69 (100.0%)	48 (100.0%)	31 (100.0%)
Western Blot	29 (42.0%)	16 (33.3%)	15 (48.4%)
Osteogenic Differentiation	21 (30.4%)	8 (16.7%)	6 (19.4%)
Alizarin Red Staining	14 (20.3%)	7 (14.6%)	7 (22.6%)
Immunofluorescence	11 (15.9%)	17 (35.4%)	11 (35.5%)
Cell Counting	10 (14.5%)	5 (10.4%)	5 (16.1%)
qPCR	7 (10.1%)	—	2 (6.5%)
RT-PCR	7 (10.1%)	9 (18.8%)	3 (9.7%)
ELISA	—	7 (14.6%)	—
Electron Microscopy	6 (8.7%)	3 (6.3%)	3 (9.7%)
Immunohistochemistry	—	5 (10.4%)	3 (9.7%)
Cell Sorting	5 (7.2%)	—	—

In the immunological applications, immunofluorescence was the second most common method after flow cytometry, used in 17 out of 48 studies (35.4%). Western blotting was employed in 16 studies (33.3%), and RT-PCR was used in 9 studies (18.8%). ELISA appeared in 7 studies (14.6%), indicating its role in quantifying cytokine levels and other soluble factors. Notably, osteogenic differentiation assays were still present in 8 studies (16.7%), suggesting some overlap with regenerative methodologies (Table 3).

The distribution of cell types investigated further highlights the prominence of DPSCs. In regenerative applications, DPSCs were studied in 39 out of 69 studies (56.5%), while in immunological applications, they appeared in 22 out of 48 studies (45.8%). When considering studies that addressed both regenerative and immunological applications, DPSCs were featured in 15 out of 31 studies (48.4%). Stem cells from SHED were also significant, appearing in 12 out of 69 regenerative studies (17.4%) and 12 out of 48 immunological studies (25.0%). This distribution reflects the versatility and therapeutic potential of these cell types across different research focuses (Table 4).

**Table 4:** Summarizes the cell types used in FC-related studies with regenerative applications, immunological applications and with both regenerative and immunological applications

Cell Type	Regenerative Applications (n = 69)	Immunological Applications (n = 48)	Both Applications (n = 31)
DPSCs	39 (56.5%)	22 (45.8%)	15 (48.4%)
PDLSCs	14 (20.3%)	11 (22.9%)	6 (19.4%)
SHED	12 (17.4%)	12 (25.0%)	9 (29.0%)
SCAP	10 (14.5%)	4 (8.3%)	4 (12.9%)
MSCs	7 (10.1%)	5 (10.4%)	3 (9.7%)
GMSCs	1 (1.4%)	—	—
ABMSCs	1 (1.4%)	—	—

Interestingly, among the 31 studies that addressed both regenerative and immunological applications, there was a notable integration of methodologies. Western blotting was used in 15 out of 31 studies (48.4%), immunofluorescence in 11 studies (35.5%), and alizarin red staining in 7 studies (22.6%) (Table 3). This overlap indicates a growing trend toward combining regenerative and immunomodulatory strategies, suggesting that researchers are increasingly recognizing the potential synergistic effects of integrating multiple therapeutic approaches in dental stem cell research.

The prominence of DPSCs, featuring in over half of the regenerative studies (56.5%) and nearly half of the immunological studies (45.8%), underscores their potential versatility in therapeutic applications. The significant presence of SHED in immunological studies (25.0%) compared to regenerative studies (17.4%) suggests a particular interest in their immunomodulatory properties.

The overlap in methodologies and cell types among studies focusing on both applications indicates a growing interdisciplinary approach in dental stem cell research as seen in. With 31 out of 86 relevant studies (36.0%) (Table 5) addressing both regenerative and immunological applications (Table 5), there's a clear trend toward integrated therapeutic strategies.

**Table 5:** The overlap of applications in relevant studies with FC

Application Type	Number of Studies	Percentage of Total Studies (n = 86)
Regenerative Only	38	44.2%
Immunological Only	17	19.8%
Both Regenerative and Immunological	31	36.0%
Total	**86**	**100.0%**

## Discussion

This systematic review provides a comprehensive analysis of the application of FC in dental stem cell research, highlighting its pivotal role in both immunological and regenerative contexts. From an initial pool of 430 PubMed-indexed articles, we systematically narrowed the selection to 284 pertinent studies using advanced natural language processing tools in line with PRISMA guidelines. Notably, 229 of these studies employed FC, emphasizing its indispensable role in the precise characterization and functional analysis of dental stem cells. Among these, 115 studies yielded significant results, forming a robust foundation for an in-depth evaluation of current methodologies and findings in the field.

### The Central Role of FC in Dental Stem Cell Characterization

The extensive use of FC in 229 out of 284 studies underscores its critical importance in the precise characterization and isolation of dental stem cells. FC enables quantitative analysis of cell surface and intracellular markers at the single-cell level, facilitating the isolation of specific stem cell subpopulations based on unique marker expressions. For instance, by identifying mesenchymal stem cell (MSC) markers such as CD73, CD90, and CD105, and excluding hematopoietic markers like CD34 and CD45 [6], researchers can isolate highly purified MSC populations from dental tissues [58].

This precision is particularly crucial given the heterogeneity of stem cells derived from various dental sources. Studies have demonstrated that DPSCs from healthy teeth and those affected by periodontitis differ in viability and differentiation potential [41]. FC aids in elucidating these differences by providing detailed profiles of surface marker expressions that may influence regenerative capabilities. For example, CD146-positive human DPSCs have been shown to promote the regeneration of dentin/pulp-like structures, indicating that specific subpopulations possess enhanced regenerative properties [26].

Moreover, FC has been instrumental in assessing how various factors influence stem cell characteristics. The impact of bioactive molecules such as melatonin on stem cell viability, proliferation, and differentiation can be quantified using FC, offering insights into how these agents might enhance regenerative therapies [16, 54]. Similarly, the influence of inflammatory cytokines on stem cell differentiation [69] and marker expression can be analyzed, which is particularly relevant since stem cells often reside in or are transplanted into inflamed tissues [88].

Given the complexity of the dental stem cell microenvironment, FC provides a powerful tool to dissect subtle changes in stem cell populations in response to different stimuli or pathological conditions. This can lead to the identification of novel markers associated with enhanced regenerative potential or immunomodulatory capacity. For example, upregulation of specific integrins upon osteogenic differentiation of human MSCs, as revealed by FC analysis, may offer targets for improving bone regeneration [10, 82].

FC offers detailed insights into dental stem cell populations. However, a standardized protocol for future research is needed, paving the way to explore the molecular mechanisms behind their immunological functions.

### Standardization of Flow Cytometry Methodologies in Dental Stem Cell Research

The reliability and reproducibility of research findings in dental stem cell studies are heavily dependent on standardized methodologies. Our comprehensive analysis reveals significant similarities in FC methodologies across various studies in dental stem cell research. This standardization is pivotal for accurate characterization, comparison of results, and ultimately, the advancement of both immunological and regenerative applications in the field.

#### Sample Preparation and Cell Types Studied

Proper sample preparation is a cornerstone of experimental consistency when working with dental MSCs. Researchers have noted that differences in preparation methods can alter the properties of MSCs, leading to inconsistent or irreproducible results [76]. For instance, in the context of dental pulp stem cells (a type of dental MSC), variations in isolation techniques and reagent use were shown to affect cell characteristics and contribute to divergent experimental outcomes [76]. Inconsistent protocols across laboratories– from tissue handling to cell expansion– have made it difficult to interpret and reproduce findings, highlighting the need for unified standards [68]. In response, international guidelines now recommend using standardized procedures for cell isolation, storage, and culture to improve reproducibility, efficacy, and safety of MSC-based experiments [76]. Ultimately, careful and consistent sample preparation minimizes “product irregularities” in MSC research, helping researchers obtain consistent, reliable data that can be replicated by others [76]. Consistent sample preparation protocols are fundamental for ensuring data reliability. The majority of the reviewed studies utilized enzymatic digestion (58.9%) with collagenase type I and/or dispase II to isolate dental mesenchymal stem cells (MSCs) from tissues such as dental pulp, periodontal ligament, and apical papilla [20, 23, 28, 33, 36, 56, 63, 66, 84, 87]. Comparative analyses between MSCs from healthy and inflamed tissues provided insights into how pathological conditions affect stem cell properties [33, 66]. For example, inflammation can influence MSC proliferation, differentiation potential, surface marker expression, and immunomodulatory capacity. PDLSCs derived from inflamed periodontal tissue exhibited higher proliferation and migration but a reduced osteogenic differentiation capacity compared to those from healthy periodontium, suggesting that a chronic inflammatory microenvironment can drive a more proliferative yet less differentiation-prone phenotype [66]. In contrast, DPSCs isolated from inflamed pulp maintained proliferation rates and multi-lineage differentiation ability similar to DPSCs from healthy pulp, indicating that not all dental MSC sources respond identically to inflammation [33]. Notably, MSCs from both healthy and diseased tissues continued to express typical mesenchymal surface markers (e.g., CD73, CD90, CD105), fulfilling standard MSC identity criteria despite the pathological context [33, 66]. However, inflammatory conditions markedly impacted the immunomodulatory function of these cells. MSCs isolated from inflamed tissue showed a diminished immunosuppressive capacity in vitro: DPSCs from irreversible pulpitis had a significantly reduced ability to suppress T-cell proliferation and lower indoleamine 2,3-dioxygenase activity compared to healthy pulp DPSCs, and similarly, periodontitis-derived PDLSCs exhibited a weaker capacity to modulate immune cell activity than their healthy counterparts [33, 66]. In co-culture systems with peripheral blood mononuclear cells (PBMCs), these differences became evident, underscoring that an inflamed microenvironment can attenuate the anti-inflammatory and immunoregulatory interactions typically exerted by dental MSCs [23, 33, 84]. Nevertheless, under non-pathological conditions, dental MSCs such as SHED display robust immunomodulatory properties. Yamaza et al. [84] demonstrated that SHED can inhibit pro-inflammatory T helper 17 cells and promote regulatory T cells, effectively helping resolve systemic inflammation. This highlights the contrast in functional potency between MSCs from healthy versus diseased tissue environments.

#### Antibody Selection and Staining Protocols

A consistent panel of antibodies was employed across studies for the identification and characterization of MSCs. Positive MSC markers included CD29, CD44, CD73, CD90, CD105, CD146, STRO- 1, and SSEA4, while negative hematopoietic markers such as CD14, CD34, CD45, and HLA-DR confirmed the absence of hematopoietic contamination [23, 28, 33, 36, 48, 57, 63, 66, 84, 85, 87]. For differentiation studies, lineage-specific markers like VE-Cadherin (CD144) for endothelial cells, alkaline phosphatase (ALP) and Runx2 for osteogenic differentiation, and peroxisome proliferator-activated receptor gamma 2 (PPARγ2) for adipogenic differentiation were assessed [37, 39, 85, 87]. Table 6 lists the most frequently used surface and identification markers.

**Table 6:** Summarizes groups of commonly used molecular markers to identify distinct cell types or lineages. The first column lists surface stem cell markers frequently used to characterize mesenchymal or other adult stem cells. The middle column contains neuronal identification markers indicative of neuronal or glial differentiation. The right column shows markers associated with immune stem cells and cell adhesion

Surface Stem Cell Markers	Neuronal identification Markers	Immune Stem Cell Markers, Cell Adhesion Markers,
CD105	Nestin	Fibronectin
CD90	ChAT	Involucrin
CD34	GAD 65/67	ITGß1
CD44	vGlut2	CD45
STRO- 1	TH	CD73
CD29	S100	
CD271	NFM	
CD166	GFAP	
CD146	ß3-Tubulin	

#### Data Analysis Techniques and Adherence to ISCT Guidelines

Flow cytometry data acquisition was performed using instruments like BD Calibur or BD LSR II, with software such as FlowJo or BD Diva utilized for analysis. Controls, including unstained cells, single-stain controls, and fluorescence-minus-one (FMO) controls, ensured accurate compensation and gating strategies [23, 28, 33, 36, 48, 57, 63, 66, 84, 85, 87]. Quantitative measurements, such as calculating the percentage of positive cells and mean fluorescence intensity (MFI) for each marker, allowed for precise characterization of cell populations.

Adherence to the International Society for Cellular Therapy (ISCT) guidelines was evident across studies, with MSCs consistently defined by their plastic adherence and characteristic immunophenotype– specifically, ≥ 95% expression of MSC markers CD73, CD90, and CD105, coupled with a lack of hematopoietic markers CD34 and CD45 (usually ≤ 2% expression to confirm minimal hematopoietic contamination). In addition, all studies confirmed the cells’ multipotency via standard tri-lineage differentiation assays: osteogenic differentiation was verified by calcium deposition (e.g., Alizarin Red S staining), adipogenic differentiation by lipid accumulation (Oil Red O staining), and chondrogenic differentiation by cartilage-matrix formation (Alcian Blue staining). Minor variations in marker expression were noted across different MSC sources– for instance, adipose-derived MSCs often exhibited transient CD34 positivity at early passages, which gradually disappeared with further culturing– but overall, all MSC populations met the ISCT criteria for surface markers and multipotency. Each study also employed rigorous validation methods to ensure compliance with these criteria, most commonly using flow cytometry to quantify surface marker expression (confirming the ≥ 95%/≤ 2% marker profile) and histological or immunocytochemical staining to demonstrate osteogenic, adipogenic, and chondrogenic differentiation, thereby conclusively verifying that the isolated cells fulfilled the standard MSC identity. [23, 28, 33, 36, 48, 57, 63, 66, 84, 85, 87]. This standardization enhances the comparability of results and ensures that MSC populations used in experiments are consistently characterized.

#### Applications of Flow Cytometry in Functional Analyses

Flow cytometry was integral not only for cell characterization but also for functional analyses across the reviewed studies. In differentiation studies, changes in marker expression were monitored over time to assess the efficiency of lineage commitment. For instance, the upregulation of VE-Cadherin during endothelial differentiation of DPSCs was quantified using flow cytometry [37, 39]. Similarly, the expression of ALP and Runx2 was assessed during osteogenic differentiation of PDLSCs [85].

For apoptosis and cell cycle analyses, standardized protocols were employed. Annexin V-FITC/propidium iodide (PI) staining was used to distinguish between live, early apoptotic, and late apoptotic or necrotic cells [56, 57, 66, 85]. PI staining, along with RNase A treatment, enabled cell cycle analysis by measuring DNA content [57]. Functional assays, such as apoptosis detection and cell cycle analysis, were standardized. Annexin V-FITC/PI staining allowed for the quantification of apoptosis rates in response to treatments like miR- 224 inhibition or exposure to exogenous factors [56, 66, 85]. Cell cycle progression under the influence of cytokines like TNF-α was evaluated using PI staining, providing insights into proliferative responses [57].

In immunomodulation studies, flow cytometry was employed to assess the impact of dental MSCs on immune cells. Co-culture experiments with lymphocytes and subsequent analysis of proliferation and activation markers elucidated the immunosuppressive capabilities of dental stem cells [23, 33, 84]. Intracellular staining for phosphorylated proteins enabled the examination of signaling pathways like JNK/MAPK and Akt/GSK- 3β, shedding light on molecular mechanisms underlying stem cell functions [57, 85].

#### Clinical Implications of Standardized Methodologies

The standardization of flow cytometry methodologies has significant clinical implications. Consistent identification and characterization of MSCs facilitate the accurate assessment of their therapeutic potential. This is particularly crucial for regenerative applications where the selection of specific subpopulations, such as CD105^ +/CD146^ + cells, may enhance tissue integration and vascularization. Standardized protocols ensure that MSCs used in clinical settings meet defined criteria for safety and efficacy.

Moreover, uniform methodologies enable reproducibility and comparability across studies, accelerating the translation of research findings into clinical applications. As dental stem cells progress toward therapeutic use, standardized flow cytometry protocols will be essential for regulatory approval and for ensuring consistent patient outcomes.

For this reason optimized multicolor immunophenotyping panels (OMIPs) have been proposed to improve standardization and reproducibility in flow cytometry analyses, a benefit that can greatly enhance dental stem cell research​

By using a peer-reviewed, pre-validated panel of multiple surface markers, OMIPs ensure that different laboratories characterize cells with the same optimized protocols and reagents, facilitating direct comparison of results across studies​. This consistency is especially valuable for identifying and characterizing dental stem cell subpopulations: an OMIP allows simultaneous multi-marker analysis, improving the resolution at which rare or functionally distinct subsets (e.g. perivascular vs. pulp stromal cells) are defined​. For example, a dental stem cell-specific OMIP could incorporate common mesenchymal stem cell markers such as CD73, CD90, CD105, and CD44, along with dental pulp stem cell markers like STRO- 1, CD146, and CD271, to comprehensively phenotype these cells. Implementing such a standardized panel would not only refine the delineation of dental stem cell subpopulations but also ensure that findings are consistently reproducible and comparable across different studies [45, 61].

### Methodological Approaches and Technical Nuances

The methodological rigor of the reviewed studies is evident in the diverse array of techniques employed alongside FACS. Western blotting and immunofluorescence were commonly used to validate protein expression and localization. Osteogenic differentiation assays and alizarin red staining provided functional evidence of mineralization and extracellular matrix deposition [67].

Cell counting and viability assays were critical for assessing cell proliferation and cytotoxicity, ensuring that observed effects were due to specific molecular interventions rather than nonspecific cell death [51]. Transfection methods, including viral vectors and lipid-based systems, facilitated the manipulation of gene expression to investigate the roles of specific genes and signaling pathways in stem cell function [77].

The integration of multiple methodological approaches strengthened the validity of the findings, allowing for comprehensive characterization of dental stem cells at both the molecular and functional levels. However, variations in experimental protocols, such as differences in antibody panels for FACS, transfection efficiency, and differentiation conditions, highlight the need for standardization to improve reproducibility and comparability across studies.

Standardization efforts should include the use of consistent marker panels for stem cell identification, standardized differentiation protocols, and uniform reporting of experimental conditions. Addressing these methodological challenges is essential for accurately interpreting cell type-specific findings, which have significant implications for therapeutic applications.

### Immunological Applications: Molecular Mechanisms and Pathways

Among the 115 studies with significant results, 48 focused on immunological applications, underscoring the substantial interest in the immunomodulatory properties of dental stem cells. These studies investigated various types of dental stem cells, including DPSCs, PDLSCs, SHED, and SCAP.

At the molecular level, dental stem cells modulate immune responses through multiple mechanisms. They secrete anti-inflammatory cytokines such as interleukin- 6 (IL- 6) [78] and transforming growth factor-beta 1 (TGF-β1) [81], which can suppress lymphocyte proliferation and promote immune tolerance. The expression of indoleamine 2,3-dioxygenase (IDO) by these cells depletes tryptophan, further inhibiting T-cell proliferation. Some studies have demonstrated that DPSCs can induce regulatory T cells (Tregs) [41] via the PD- 1/PD-L1 pathway, contributing to immunosuppression and tolerance, which has potential therapeutic implications for autoimmune diseases like type 1 diabetes [5, 7].

Furthermore, the secretion of extracellular vesicles and exosomes containing microRNAs [90] (e.g., miR- 146a, miR- 155, miR- 758 - 5p) by dental stem cells plays a critical role in immunomodulation [24, 56, 90]. These microRNAs can modulate the phenotype of dendritic cells and macrophages, skewing them towards an anti-inflammatory M2 phenotype [30, 90]. For instance, exosomes from SHED have been shown to promote recovery after traumatic brain injury by shifting microglial polarization from the pro-inflammatory M1 phenotype to the anti-inflammatory M2 phenotype in rat models [40]. Similarly, SCAP have demonstrated potential immunomodulatory effects on Treg conversion, aiding in tissue regeneration [41].

Interestingly, while MSCs from dental tissues generally exhibit strong immunomodulatory capacities, some studies report that DPSCs derived from inflamed dental pulp or from certain donors may exhibit impaired immunomodulatory function *in vitro* [33]. This suggests that the microenvironment and donor characteristics can influence the immunomodulatory properties of dental stem cells. Additionally, stem cells from human deciduous teeth have been reported to correct immune imbalances in allergic rhinitis, indicating their potential in treating allergic conditions [19].

Additionally, the expression of transcription factors like retinoic acid-related orphan receptor beta (RORβ) in inflamed DPSCs is linked to changes in macrophage phenotype, shedding light on the immunomodulatory effects of dental stem cells [30].

Grasping these immunomodulatory mechanisms expands our understanding of how dental stem cells regulate the immune system. It also lays the groundwork for exploring their regenerative applications, especially in osteogenic differentiation and tissue engineering, which we address in the next section.

### Regenerative Applications: Odontogenic and odontogenic Differentiation and Tissue Engineering

A total of 69 studies investigated regenerative applications, with a strong emphasis on dental pulp regeneration and tissue engineering. FC was instrumental in isolating subpopulations with enhanced regenerative potential, such as STRO- 1^ + [32] and CD146^ + cells [26, 35, 37, 39]. These markers are associated with perivascular cells, which have been shown to possess superior odontogenic and angiogenic capabilities [10, 26].

Molecular assays, including reverse transcription-polymerase chain reaction (RT-PCR) and quantitative PCR (qPCR), were used to quantify the expression of odontogenic markers such as dentin sialophosphoprotein (DSPP) [10, 25], dentin matrix protein 1 (DMP1) [44], alkaline phosphatase (ALP) [24], and osteocalcin (BGLAP) [44]. The upregulation of these genes indicates the commitment of stem cells toward the odontoblastic lineage, which is crucial for dental pulp regeneration [55]. Western blot analyses further confirmed the protein expression levels of these markers, providing insights into the translational regulation during differentiation [55].

Several studies employed transfection techniques to modulate gene expression, introducing odontogenic factors like bone morphogenetic proteins (BMP- 2 and BMP- 7) [35, 89] and signaling components of pathways such as Notch1 [86] and Wnt/β-catenin [80]. Activation of the Notch1 pathway, for instance, was shown to promote the proliferation and differentiation of dental pulp stem cells via KAT2 A-mediated succinylation modification [86]. Similarly, modulation of the Wnt/β-catenin pathway influenced odontogenic differentiation, with inhibition leading to reduced differentiation potential [80].

The inflammatory microenvironment was found to significantly affect the regenerative capabilities of dental pulp stem cells [51]. Studies demonstrated that moderate pulpitis conditions enhance the osteo/odontogenic potential of DPSCs by inducing autophagy [88]. Conversely, stimulation with inflammatory cytokines altered the differentiation ability of DPSCs, suggesting a complex interplay between inflammation and regeneration.

Natural compounds such as baicalin were investigated for their ability to enhance the differentiation of inflammatory dental pulp stem cells by inhibiting NF-κB and Wnt/β-catenin signaling pathways [80]. Additionally, the use of small molecules and growth factors, including BMPs and vascular endothelial growth factors (VEGFs), was explored to induce DPSCs into various lineages, such as endothelial and neuronal cells, expanding their potential applications in regenerative medicine [20].

However, the regenerative potential of dental stem cells is intricately linked to their immunomodulatory properties, suggesting that a deeper exploration of the interplay between immunological factors and regeneration is essential, as discussed in the subsequent section.

### Interconnectedness of Immunomodulation and Regeneration

The convergence of studies addressing both immunological and regenerative applications underscores the intricate interplay between the immune environment and the regenerative capacity of dental stem cells. Pro-inflammatory cytokines such as tumor necrosis factor-alpha (TNF-α) [64] and interleukin- 1 beta (IL- 1β) [64] significantly influence stem cell differentiation by activating signaling pathways like nuclear factor kappa-light-chain-enhancer of activated B cells (NF-κB) and mitogen-activated protein kinases (MAPKs) [15], which can either promote or inhibit osteogenesis depending on the context.

For instance, baicalin has been shown to enhance the differentiation of inflammatory dental pulp stem cells by inhibiting NF-κB and Wnt/β-catenin signaling pathways, highlighting the potential of targeting these pathways to improve regenerative outcomes [38]. Functional analyses revealed that exposure to an inflammatory milieu alters the expression of surface markers and the differentiation potential of stem cells. Knockdown of transient receptor potential melastatin 2 (TRPM2) in PDLSCs promoted their differentiation by modulating the NF-κB/NLRP3 pathway, suggesting that manipulating specific inflammatory signaling pathways can enhance regeneration [79].

Additionally, lipopolysaccharide (LPS)-induced pro-inflammatory cytokines did not compromise the osteogenic potential of PDLSCs, indicating resilience in their regenerative capabilities despite inflammatory conditions [4]. Preconditioning stem cells with immunomodulatory factors like interferon-gamma (IFN-γ) enhanced their immunosuppressive properties by upregulating IDO and PD-L1 while also modulating differentiation pathways [73].

Studies also demonstrated that SCAP can promote regulatory Treg conversion, which is beneficial for tissue regeneration [41]. However, mesenchymal stem cells derived from human dental pulp exhibited impaired immunomodulatory capacity under certain conditions, emphasizing the critical role of the immunological milieu [41].

These findings highlight the importance of considering the immune environment in regenerative therapies and suggest that targeting immunomodulatory pathways may enhance the therapeutic efficacy of dental stem cells.

### Cell Type-Specific Findings and Implications

DPSCs were the most studied cell type, likely due to their accessibility and potent regenerative capabilities. FC analysis revealed that DPSCs express high levels of angiogenic factors like vascular endothelial growth factor (VEGF) and angiopoietin- 1, contributing to neovascularization in regenerative applications [20, 70]. PDLSCs and SHED also demonstrated significant osteogenic and immunomodulatory properties, with SHED exhibiting higher proliferative rates and expression of pluripotency markers such as NANOG and OCT4 [60, 71].

SCAP were studied extensively, highlighting their role in root development and potential in endodontic regeneration. FC analysis of SCAP revealed the expression of neural crest markers like Nestin and p75 NTR, suggesting a multipotent phenotype capable of differentiating into neuronal and glial cells, which could be beneficial for neural tissue engineering [72].

These cell type-specific findings emphasize the importance of selecting appropriate stem cell sources for different therapeutic applications. Understanding the unique properties and potentials of each dental stem cell type can inform the development of targeted regenerative strategies. Nevertheless, despite these promising insights, certain limitations exist in current research that must be addressed to fully harness the potential of these cell types.

### Limitations and Future Perspectives

While the data provide valuable insights, several limitations must be acknowledged. The heterogeneity of study designs, cell sources, and analytical methods complicates direct comparisons and meta-analyses. The reliance on *in vitro* studies limits the extrapolation of findings to *in vivo* contexts. Moreover, variations in FC protocols, such as differences in antibody panels, fluorochrome selection, and gating strategies, can affect the reproducibility and interpretation of results.

Future research should aim to standardize FC methodologies, including the use of appropriate controls and consistent marker panels, to enhance data quality. The integration of advanced technologies such as single-cell RNA sequencing (scRNA-seq) with FC could provide deeper insights into the heterogeneity of dental stem cell populations and identify novel subpopulations with unique functional properties.

*In vivo* studies and clinical trials are necessary to validate the therapeutic potential of dental stem cells. Investigations into scaffold materials, three-dimensional culture systems, and bioreactors could improve cell survival, integration, and function after transplantation. Additionally, understanding the long-term effects of genetic modifications and ensuring the safety of stem cell therapies remain paramount.

By overcoming these challenges, we can pave the way for significant clinical implications and translational potential in dental stem cell research.

### Clinical Implications and Translational Potential

The molecular insights gained from this review have significant clinical implications. The immunomodulatory properties of dental stem cells could be harnessed to treat autoimmune diseases, inflammatory conditions, and transplant rejection. Their regenerative capabilities hold promise for repairing dental tissues, craniofacial defects, and even bone and neural tissues beyond the oral cavity.

The use of FC to isolate specific stem cell subpopulations enhances the precision of cell-based therapies. For example, selecting CD105^ +/CD146^ + cells could improve angiogenesis and tissue integration in regenerative procedures [26, 35, 58]. Moreover, understanding the molecular pathways that govern stem cell behavior allows for the development of targeted pharmacological agents or gene therapies to enhance desired outcomes.

Translating these findings into clinical practice will require rigorous preclinical studies and well-designed clinical trials to assess safety, efficacy, and long-term outcomes. Collaboration between researchers, clinicians, and regulatory agencies will be essential to navigate the challenges and accelerate the development of effective stem cell-based therapies for dental and systemic applications.

#### Expanding Applications: Maternal Oral Health During Pregnancy

An emerging and significant application of dental stem cell research lies in promoting oral health during pregnancy, a period characterized by hormonal fluctuations that can exacerbate oral conditions like gingivitis and periodontitis. These conditions not only affect maternal well-being but can also have adverse effects on fetal development, including risks of preterm birth and low birth weight [12]. Leveraging the immunomodulatory and regenerative properties of dental stem cells offers a promising avenue for therapeutic interventions tailored to pregnant women.

Recent studies, such as the concise review by Meto et al. [46, 46], have highlighted the potential of dental stem cells, particularly those derived from dental pulp and periodontal ligaments, in addressing pregnancy-induced oral health issues [46]. The review emphasizes how dental stem cells can modulate immune responses and promote tissue regeneration in the unique physiological context of pregnancy. For instance, the secretion of anti-inflammatory cytokines and growth factors by these cells can mitigate inflammation without adversely affecting fetal health. Incorporating dental stem cells into treatment strategies could enhance periodontal therapy outcomes during pregnancy, ultimately benefiting both maternal and fetal health. This integration of dental stem cell applications into broader clinical contexts underscores the translational potential of our current findings and aligns with the growing emphasis on personalized and safe therapeutic approaches in maternal healthcare.

#### Novel Horizons: Transdifferentiation Potential and Systemic Regeneration

Building upon the versatility of dental stem cells discussed in previous sections, recent studies have unveiled their remarkable potential to transdifferentiate into non-dental cell types, offering promising avenues for systemic regenerative therapies. This transdifferentiation capability not only underscores the plasticity of dental stem cells but also highlights the pivotal role of FC in isolating and characterizing specific subpopulations with enhanced differentiation potential.

A notable study by Abuarqoub et al. [1] explored the differentiation of SCAP into functional insulin-producing beta-like cells. Utilizing FC to confirm the upregulation of definitive endoderm markers FOXA2 and SOX17, they successfully guided SCAP through pancreatic lineage commitment. The differentiated cells formed islet-like clusters that stained positive with diphenylthiocarbazone (DTZ) and exhibited significant secretion of insulin and C-peptide, indicating functional maturity akin to pancreatic beta cells. This breakthrough suggests that SCAP, accessible through minimally invasive procedures, could serve as a novel cell source for treating type 1 diabetes mellitus by replenishing the depleted beta-cell population.

Similarly, Tsai et al. [71] investigated the potential of SHEDs to differentiate into corneal epithelial-like cells. By co-culturing SHED with immortalized human corneal epithelial cells in a transwell system, they observed upregulation of epithelial markers cytokeratin 3 (CK3) and cytokeratin 19 (CK19), confirmed through immunofluorescence and RT-PCR. FC analysis revealed that SHED express mesenchymal stem cell markers along with pluripotency genes NANOG and OCT- 4, highlighting their versatility. This finding opens new possibilities for using SHED in ocular surface regeneration, providing alternative therapies for patients with corneal epithelial defects.

These studies reinforce the interconnectedness of immunomodulation and regeneration discussed earlier. The ability of dental stem cells to transdifferentiate into various cell types is intricately linked to their immunomodulatory properties, facilitating integration and function in non-native tissues. Moreover, the methodological approaches employed, particularly the use of FC for precise cell isolation and characterization, align with the technical nuances highlighted in our systematic review.

The transdifferentiation potential of dental stem cells accentuates the importance of cell type-specific findings and implications. Understanding the unique properties of SCAP and SHED enables the development of targeted regenerative strategies for systemic conditions. These findings also underscore the limitations and future perspectives outlined previously, emphasizing the need for standardized methodologies and *in vivo* studies to validate therapeutic potential.

## Conclusion

This systematic review demonstrates that flow cytometry plays a crucial role in identifying and characterizing immunomodulatory markers in dental stem cells, thereby enhancing their potential applications in regenerative medicine. By enabling precise analysis of immunoregulatory molecules such as PD-L1, IDO, and TGF-β1, flow cytometry facilitates a deeper understanding of how dental stem cells interact with the immune system. This understanding is essential for developing targeted therapies that harness the immunomodulatory properties of dental stem cells. Standardization of flow cytometry methodologies is imperative to overcome technical challenges and ensure reproducibility. Future research should focus on refining these techniques and exploring the clinical implications, ultimately contributing to the development of effective dental stem cell-based therapies for patients with dental and systemic diseases.

## Data Availability

The data supporting the reported results of this study are openly available and registered in the Open Science Framework (OSF) repository at https://osf.io/srm35/. All datasets analyzed during the study can be accessed through this link.

## Abbreviations

DPSCs: Dental Pulp Stem Cells
SHED: Stem Cells from Human Exfoliated Deciduous Teeth
PDLSCs: Periodontal Ligament Stem Cells
SCAP: Stem Cells from the Apical Papilla
GMSCs: Gingival Mesenchymal Stem Cells
MSCs: Mesenchymal Stem Cells
FACS: Fluorescence-Activated Cell Sorting
CD: Cluster of Differentiation (e.g., CD34, CD45, CD73, CD90, CD105)
IDO: Indoleamine 2,3-Dioxygenase
PD-L1: Programmed Death-Ligand 1
TGF-β1: Transforming Growth Factor Beta 1
qPCR: Quantitative Polymerase Chain Reaction
RT-PCR: Reverse Transcription Polymerase Chain Reaction
NF-κB: Nuclear Factor Kappa B

## References

[1] Abuarqoub, D., Adwan, S., Zaza, R., Wehaibi, S., Aslam, N., Jafar, H., Qinnah, N., & Awidi, A. (2023). Effective generation of functional pancreatic β cells from human-derived dental stem cells of apical papilla and bone-marrow-derived stem cells: A comparative study. *Pharmaceuticals*, *16*(5), 649. 10.3390/ph16050649 37242432 10.3390/ph16050649 PMC10222296

[2] Abuarqoub, D., Aslam, N., Almajali, B., Shajrawi, L., Jafar, H., & Awidi, A. (2020). Neuro-regenerative potential of dental stem cells: A concise review. *Cell and Tissue Research*, *382*(2), 267–279. 10.1007/s00441-020-03255-0 32725424 10.1007/s00441-020-03255-0

[3] Abuarqoub, D., Aslam, N., Zaza, R., Jafar, H., Zalloum, S., Atoom, R., Alshaer, W., Al-Mrahleh, M., & Awidi, A. (2022). The immunomodulatory and regenerative effect of biodentine™ on human THP-1 cells and dental pulp stem cells In Vitro study. *BioMed Research International*, *2022*, 2656784. 10.1155/2022/2656784 36093401 10.1155/2022/2656784 PMC9462999

[4] Albiero, M. L., Amorim, B. R., Casati, M. Z., Sallum, E. A., Nociti, F. H. J., & Silvério, K. G. (2017). Osteogenic potential of periodontal ligament stem cells are unaffected after exposure to lipopolysaccharides. *Brazilian Oral Research*, *31*, e17. 10.1590/1807-3107BOR-2017.vol31.0017 28146221 10.1590/1807-3107BOR-2017.vol31.0017

[5] Alipour, R., Sadeghi, F., Hashemi-Beni, B., Zarkesh-Esfahani, S. H., Heydari, F., Mousavi, S. B., Adib, M., Narimani, M., & Esmaeili, N. (2010). Phenotypic characterizations and comparison of adult dental stem cells with adipose-derived stem cells. *International Journal of Preventive Medicine*,* 1*(3), 164–171. https://www.ncbi.nlm.nih.gov/pmc/articles/PMC3075526/pdf/IJPVM-1-164.pdf PMC3075526 21566786

[6] Asgharian-Rezaee, M., Alipour-Farmad, R., & Tayarani-Najaran, Z. (2020). Comparison of osteogenic potential of phenytoin with dexamethasone in cultured dental pulp stem cells. *Reports of Biochemistry and Molecular Biology*, *9*(3), 331–337. 10.29252/rbmb.9.3.331 33649727 10.29252/rbmb.9.3.331 PMC7816787

[7] Ashour, L., Al Habashneh, R. A., Al-Mrahelh, M. M., Abuarqoub, D., Khader, Y. S., Jafar, H., & Awidi, A. S. (2020). The modulation of mature dendritic cells from patients with type 1 diabetes using human periodontal ligament stem cells. An in-vitro study. *Journal of Diabetes & Metabolic Disorders*, *19*(2), 1037–1044. 10.1007/s40200-020-00602-4 33520821 10.1007/s40200-020-00602-4 PMC7843723

[8] Bai, Y., Cheng, X., Liu, X., Guo, Q., Wang, Z., Fu, Y., He, W., & Yu, Q. (2023). Transforming growth factor-β1 promotes early odontoblastic differentiation of dental pulp stem cells via activating AKT, Erk1/2 and p38 MAPK pathways. *Journal of Dental Sciences*, *18*(1), 87–94. 10.1016/j.jds.2022.06.027 36643229 10.1016/j.jds.2022.06.027 PMC9831829

[9] Bakopoulou, A., & About, I. (2016). Stem cells of dental origin: Current research trends and key milestones towards clinical application. *Stem Cells International*, *2016*, 4209891. 10.1155/2016/4209891 27818690 10.1155/2016/4209891 PMC5081960

[10] Bakopoulou, A., Leyhausen, G., Volk, J., Koidis, P., & Geurtsen, W. (2013). Comparative characterization of STRO-1(neg)/CD146(pos) and STRO-1(pos)/CD146(pos) apical papilla stem cells enriched with flow cytometry. *Archives of Oral Biology*, *58*(10), 1556–1568. 10.1016/j.archoralbio.2013.06.018 23871383 10.1016/j.archoralbio.2013.06.018

[11] Bergamo, M. T., Zhang, Z., Oliveira, T. M., & Nör, J. E. (2021). VEGFR1 primes a unique cohort of dental pulp stem cells for vasculogenic differentiation. *European Cells and Materials*, *41*, 332–344. 10.22203/eCM.v041a21 33724439 10.22203/eCM.v041a21 PMC8561749

[12] Bobetsis, Y. A., Graziani, F., Gürsoy, M., & Madianos, P. N. (2020). Periodontal disease and adverse pregnancy outcomes. *Periodontology 2000*, *83*(1), 154–174. 10.1111/prd.12294 32385871 10.1111/prd.12294

[13] Bojic, S., Volarevic, V., Ljujic, B., & Stojkovic, M. (2014). Dental stem cells–characteristics and potential. *Histology & Histopathology*, *29*(6), 699–706. 10.14670/hh-29.699 24446280 10.14670/HH-29.699

[14] Capparè, P., Tetè, G., Sberna, M. T., & Panina-Bordignon, P. (2020). The emerging role of stem cells in regenerative dentistry. *Current Gene Therapy*, *20*(4), 259–268. 10.2174/1566523220999200818115803 32811413 10.2174/1566523220999200818115803

[15] Chan, Y. H., Lee, Y. C., Hung, C. Y., Yang, P. J., Lai, P. C., & Feng, S. W. (2021). Three-dimensional spheroid culture enhances multipotent differentiation and stemness capacities of human dental pulp-derived mesenchymal stem cells by modulating MAPK and NF-kB signaling pathways. *Stem Cell Reviews and Reports*, *17*(5), 1810–1826. 10.1007/s12015-021-10172-4 33893620 10.1007/s12015-021-10172-4

[16] Cvikl, B., Hess, S. C., Miron, R. J., Agis, H., Bosshardt, D., Attin, T., Schmidlin, P. R., & Lussi, A. (2017). Response of human dental pulp cells to a silver-containing PLGA/TCP-nanofabric as a potential antibacterial regenerative pulp-capping material. *BMC Oral Health*, *17*(1), 57. 10.1186/s12903-017-0348-7 28241819 10.1186/s12903-017-0348-7 PMC5327548

[17] Cvikl, B., Lussi, A., Moritz, A., Sawada, K., & Gruber, R. (2016). Differential inflammatory response of dental pulp explants and fibroblasts to saliva. *International Endodontic Journal*, *49*(7), 655–662. 10.1111/iej.12493 26114806 10.1111/iej.12493

[18] d’Aquino, R., Graziano, A., Sampaolesi, M., Laino, G., Pirozzi, G., De Rosa, A., & Papaccio, G. (2007). Human postnatal dental pulp cells co-differentiate into osteoblasts and endotheliocytes: A pivotal synergy leading to adult bone tissue formation. *Cell Death and Differentiation*, *14*(6), 1162–1171. 10.1038/sj.cdd.4402121 17347663 10.1038/sj.cdd.4402121

[19] Dai, Y. Y., Ni, S. Y., Ma, K., Ma, Y. S., Wang, Z. S., & Zhao, X. L. (2019). Stem cells from human exfoliated deciduous teeth correct the immune imbalance of allergic rhinitis via Treg cells in vivo and in vitro. *Stem Cell Research & Therapy*, *10*(1), 39. 10.1186/s13287-019-1134-z 30670101 10.1186/s13287-019-1134-z PMC6341645

[20] de Cara, S., Origassa, C. S. T., de Sá Silva, F., Moreira, M., de Almeida, D. C., Pedroni, A. C. F., Carvalho, G. L., Cury, D. P., Câmara, N. O. S., & Marques, M. M. (2019). Angiogenic properties of dental pulp stem cells conditioned medium on endothelial cells in vitro and in rodent orthotopic dental pulp regeneration. *Heliyon*, *5*(4), e01560. 10.1016/j.heliyon.2019.e01560 31183428 10.1016/j.heliyon.2019.e01560 PMC6488540

[21] De la Rosa-Ruiz, M. D. P., Álvarez-Pérez, M. A., Cortés-Morales, V. A., Monroy-García, A., Mayani, H., Fragoso-González, G., Caballero-Chacón, S., Diaz, D., Candanedo-González, F., & Montesinos, J. J. (2019). Mesenchymal stem/stromal cells derived from dental tissues: A comparative in vitro evaluation of their immunoregulatory properties against T cells. *Cells*,* 8*(12). 10.3390/cells8121491 10.3390/cells8121491 PMC6953107 31766697

[22] Di Tinco, R., Bertani, G., Pisciotta, A., Bertoni, L., Pignatti, E., Maccaferri, M., Bertacchini, J., Sena, P., Vallarola, A., Tupler, R., Croci, S., Bonacini, M., Salvarani, C., & Carnevale, G. (2021). Role of PD-L1 in licensing immunoregulatory function of dental pulp mesenchymal stem cells. *Stem Cell Research & Therapy*, *12*(1), 598. 10.1186/s13287-021-02664-4 34863286 10.1186/s13287-021-02664-4 PMC8643194

[23] Ding, G., Niu, J., & Liu, Y. (2015). Dental pulp stem cells suppress the proliferation of lymphocytes via transforming growth factor-β1. *Human Cell*, *28*(2), 81–90. 10.1007/s13577-014-0106-y 25605036 10.1007/s13577-014-0106-y

[24] Du, L., Cao, W. J., Tian, Y., & Wang, Y. M. (2018). MicroRNA-125b regulates osteogenic differentiation of human periodontal ligament stem cells. *Shanghai Kou Qiang Yi Xue*, *27*(1), 11–17. 29946633

[25] Du, Q., Cao, L., Liu, Y., Pang, C., Wu, S., Zheng, L., Jiang, W., Na, X., Yu, J., Wang, S., Zhu, X., & Yang, J. (2021). Phenotype and molecular characterizations of a family with dentinogenesis imperfecta shields type II with a novel DSPP mutation. *Annals of Translational Medicine*, *9*(22), 1672. 10.21037/atm-21-5369 34988181 10.21037/atm-21-5369 PMC8667123

[26] Ebrahimi Dastgurdi, M., Ejeian, F., Nematollahi, M., Motaghi, A., & Nasr-Esfahani, M. H. (2018). Comparison of two digestion strategies on characteristics and differentiation potential of human dental pulp stem cells. *Archives of Oral Biology*, *93*, 74–79. 10.1016/j.archoralbio.2018.05.008 29852380 10.1016/j.archoralbio.2018.05.008

[27] Fageeh, H. N. (2021). Preliminary evaluation of proliferation, wound healing properties, osteogenic and chondrogenic potential of dental pulp stem cells obtained from healthy and periodontitis affected teeth. *Cells*, *10*(8). 10.3390/cells10082118 10.3390/cells10082118 PMC8393753 34440887

[28] Fracaro, L., Hochuli, A. H. D., Selenko, A. H., Capriglione, L. G. A., Brofman, P. R. S., & Senegaglia, A. C. (2023). Mesenchymal stromal cells derived from exfoliated deciduous teeth express neuronal markers before differentiation induction. *Journal of Applied Oral Science*, *31*, e20220489. 10.1590/1678-7757-2022-0489 37075387 10.1590/1678-7757-2022-0489 PMC10118381

[29] Fracaro, L., Senegaglia, A. C., Herai, R. H., Leitolis, A., Boldrini-Leite, L. M., Rebelatto, C. L. K., Travers, P. J., Brofman, P. R. S., & Correa, A. (2020). The expression profile of dental pulp-derived stromal cells supports their limited capacity to differentiate into adipogenic cells. *International Journal of Molecular Sciences*, *21*(8). 10.3390/ijms21082753 10.3390/ijms21082753 PMC7215853 32326648

[30] Gopinath, V. K., Soumya, S., & Mohammad, M. G. (2021). Ror β expression in activated macrophages and dental pulp stem cells. *International Endodontic Journal*, *54*(3), 388–398. 10.1111/iej.13431 33075145 10.1111/iej.13431

[31] Guo, H., Zhao, W., Liu, A., Wu, M., Shuai, Y., Li, B., Huang, X., Liu, X., Yang, X., Guo, X., Xuan, K., & Jin, Y. (2020). SHED promote angiogenesis in stem cell-mediated dental pulp regeneration. *Biochemical and Biophysical Research Communications*, *529*(4), 1158–1164. 10.1016/j.bbrc.2020.06.151 32819580 10.1016/j.bbrc.2020.06.151

[32] Gurel Pekozer, G., Ramazanoglu, M., Schlegel, K. A., Kok, F. N., & Torun Kose, G. (2018). Role of STRO-1 sorting of porcine dental germ stem cells in dental stem cell-mediated bone tissue engineering. *Artificial Cells, Nanomedicine, and Biotechnology*, *46*(3), 607–618. 10.1080/21691401.2017.1332637 10.1080/21691401.2017.1332637 28562085

[33] Inostroza, C., Vega-Letter, A. M., Brizuela, C., Castrillón, L., Saint Jean, N., Duran, C. M., & Carrión, F. (2020). Mesenchymal stem cells derived from human inflamed dental pulp exhibit impaired immunomodulatory capacity in vitro. *Journal of Endodontia*, *46*(8), 1091-1098.e1092. 10.1016/j.joen.2020.05.003 10.1016/j.joen.2020.05.003 32422164

[34] Kok, Z. Y., Alaidaroos, N. Y. A., Alraies, A., Colombo, J. S., Davies, L. C., Waddington, R. J., Sloan, A. J., & Moseley, R. (2022). Dental pulp stem cell heterogeneity: Finding superior quality “Needles” in a dental pulpal “Haystack” for regenerative medicine-based applications. *Stem Cells Int*, *2022*, 9127074. 10.1155/2022/9127074 35027930 10.1155/2022/9127074 PMC8752304

[35] Kunimatsu, R., Rikitake, K., Yoshimi, Y., Putranti, N. A. R., Hayashi, Y., & Tanimoto, K. (2023). Bone differentiation ability of CD146-positive stem cells from human exfoliated deciduous teeth. *International Journal of Molecular Sciences*, *24*(4). 10.3390/ijms24044048 10.3390/ijms24044048 PMC9964331 36835460

[36] Lee, U. L., Jeon, S. H., Park, J. Y., & Choung, P. H. (2011). Effect of platelet-rich plasma on dental stem cells derived from human impacted third molars. *Regenerative Medicine*, *6*(1), 67–79. 10.2217/rme.10.96 10.2217/rme.10.96 21175288

[37] Li, J., Zhu, Y., Li, N., Wu, T., Zheng, X., Heng, B. C., Zou, D., & Xu, J. (2021). Upregulation of ETV2 Expression Promotes Endothelial Differentiation of Human Dental Pulp Stem Cells. *Cell Transplantation*, *30*, 963689720978739. 10.1177/0963689720978739 33522307 10.1177/0963689720978739 PMC7863555

[38] Li, M., Wang, Y., Xue, J., Xu, Q., Zhang, Y., Liu, J., Xu, H., Guan, Z., Bian, C., Zhang, G., & Yu, Y. (2023). Baicalin can enhance odonto/osteogenic differentiation of inflammatory dental pulp stem cells by inhibiting the NF-κB and β-catenin/Wnt signaling pathways. *Molecular Biology Reports*, *50*(5), 4435–4446. 10.1007/s11033-023-08398-1 37009956 10.1007/s11033-023-08398-1 PMC10068215

[39] Li, X. Y., Zheng, L., Ma, P. T., & Zhang, Y. X. (2021). Effect of enamel matrix protein on osteogenic and adipogenic differentiation of dental pulp stem cells of deciduous teeth through miR-32. *Shanghai Kou Qiang Yi Xue*, *30*(4), 367–373. 34693428

[40] Li, Y., Yang, Y. Y., Ren, J. L., Xu, F., Chen, F. M., & Li, A. (2017). Exosomes secreted by stem cells from human exfoliated deciduous teeth contribute to functional recovery after traumatic brain injury by shifting microglia M1/M2 polarization in rats. *Stem Cell Research & Therapy*, *8*(1), 198. 10.1186/s13287-017-0648-5 28962585 10.1186/s13287-017-0648-5 PMC5622448

[41] Liu, X. M., Liu, Y., Yu, S., Jiang, L. M., Song, B., & Chen, X. (2019). Potential immunomodulatory effects of stem cells from the apical papilla on Treg conversion in tissue regeneration for regenerative endodontic treatment. *International Endodontic Journal*, *52*(12), 1758–1767. 10.1111/iej.13197 31378943 10.1111/iej.13197

[42] Louvrier, A., Euvrard, E., Nicod, L., Rolin, G., Gindraux, F., Pazart, L., Houdayer, C., Risold, P. Y., Meyer, F., & Meyer, C. (2018). Odontoblastic differentiation of dental pulp stem cells from healthy and carious teeth on an original PCL-based 3D scaffold. *International Endodontic Journal*, *51*(Suppl 4), e252–e263. 10.1111/iej.12746 28109162 10.1111/iej.12746

[43] Luke, A. M., Patnaik, R., Kuriadom, S., Abu-Fanas, S., Mathew, S., & Shetty, K. P. (2020). Human dental pulp stem cells differentiation to neural cells, osteocytes and adipocytes-An in vitro study. *Heliyon*, *6*(1), e03054. 10.1016/j.heliyon.2019.e03054 32042932 10.1016/j.heliyon.2019.e03054 PMC7002807

[44] Machla, F., Sokolova, V., Platania, V., Prymak, O., Kostka, K., Kruse, B., Agrymakis, M., Pasadaki, S., Kritis, A., Alpantaki, K., Vidaki, M., Chatzinikolaidou, M., Epple, M., & Bakopoulou, A. (2023). Tissue engineering at the dentin-pulp interface using human treated dentin scaffolds conditioned with DMP1 or BMP2 plasmid DNA-carrying calcium phosphate nanoparticles. *Acta Biomaterialia*, *159*, 156–172. 10.1016/j.actbio.2023.01.044 36708852 10.1016/j.actbio.2023.01.044

[45] Mahnke, Y., Chattopadhyay, P., & Roederer, M. (2010). Publication of optimized multicolor immunofluorescence panels. *Cytometry Part A*, *77A*(9), 814–818. 10.1002/cyto.a.20916 10.1002/cyto.a.20916 20722004

[46] Meto, A., Sula, A., Peppoloni, S., Meto, A., & Blasi, E. (2024). Leveraging dental stem cells for oral health during pregnancy: A concise review. *Dentistry Journal*, *12*(5), 127. https://www.mdpi.com/2304-6767/12/5/127 10.3390/dj12050127 PMC11120089 38786525

[47] Min, Q., Yang, L., Tian, H., Tang, L., Xiao, Z., & Shen, J. (2023). Immunomodulatory mechanism and potential application of dental pulp-derived stem cells in immune-mediated diseases. *International Journal of Molecular Sciences*, *24*(9). 10.3390/ijms24098068 10.3390/ijms24098068 PMC10178746 37175774

[48] Mohd Nor, N. H., Berahim, Z., Azlina, A., & Kannan, T. P. (2019). Identification of novel fibroblast-like cells from stem cells from human exfoliated deciduous teeth. *Clinical Oral Investigations*, *23*(11), 3959–3966. 10.1007/s00784-019-02827-x 30847574 10.1007/s00784-019-02827-x

[49] Mrozik, K. M., Wada, N., Marino, V., Richter, W., Shi, S., Wheeler, D. L., Gronthos, S., & Bartold, P. M. (2013). Regeneration of periodontal tissues using allogeneic periodontal ligament stem cells in an ovine model. *Regenerative Medicine*, *8*(6), 711–723. 10.2217/rme.13.66 24147527 10.2217/rme.13.66

[50] Nakamura, S., Yamada, Y., Katagiri, W., Sugito, T., Ito, K., & Ueda, M. (2009). Stem cell proliferation pathways comparison between human exfoliated deciduous teeth and dental pulp stem cells by gene expression profile from promising dental pulp. *Journal of Endodontia*, *35*(11), 1536–1542. 10.1016/j.joen.2009.07.024 10.1016/j.joen.2009.07.024 19840643

[51] Ning, T., Shao, J., Zhang, X., Luo, X., Huang, X., Wu, H., Xu, S., Wu, B., & Ma, D. (2020). Ageing affects the proliferation and mineralization of rat dental pulp stem cells under inflammatory conditions. *International Endodontic Journal*, *53*(1), 72–83. 10.1111/iej.13205 31419325 10.1111/iej.13205

[52] Nuti, N., Corallo, C., Chan, B. M., Ferrari, M., & Gerami-Naini, B. (2016). Multipotent differentiation of human dental pulp stem cells: A literature review. *Stem Cell Reviews and Reports*, *12*(5), 511–523. 10.1007/s12015-016-9661-9 27240827 10.1007/s12015-016-9661-9

[53] Page, M. J., McKenzie, J. E., Bossuyt, P. M., Boutron, I., Hoffmann, T. C., Mulrow, C. D., Shamseer, L., Tetzlaff, J. M., Akl, E. A., Brennan, S. E., Chou, R., Glanville, J., Grimshaw, J. M., Hróbjartsson, A., Lalu, M. M., Li, T., Loder, E. W., Mayo-Wilson, E., McDonald, S., & Moher, D. (2021). The PRISMA 2020 statement: An updated guideline for reporting systematic reviews. *BMJ*, *372*, n71. 10.1136/bmj.n71 33782057 10.1136/bmj.n71 PMC8005924

[54] Patil, S., Alamoudi, A., Zidane, B., Alzahrani, K. J., Alzahrani, F. M., Banjer, H. J., Reda, R., Balaji, T. M., Bhandi, S., Raj, A. T., & Testarelli, L. (2022). Dose-dependent effects of melatonin on the viability, proliferation, and differentiation of dental pulp stem cells (DPSCs). *Journal of Personalized Medicine*, *12*(10). 10.3390/jpm12101620 10.3390/jpm12101620 PMC9605259 36294759

[55] Pawar, M., Pawar, V., Thete, S. G., Dutta, S. D., Sadan, P. P., Maria, R., & Kulkarni, D. (2021). Enhancement of odontoblastic differentiation of stem cells from exfoliated deciduous tooth using N-acetylcysteine-an in vitro study. *The Journal of Contemporary Dental Practice*, *22*(8), 882–889. 34753839

[56] Qiao, W., Li, D., Shi, Q., Wang, H., Wang, H., & Guo, J. (2020). miR-224-5p protects dental pulp stem cells from apoptosis by targeting Rac1. *Experimental and Therapeutic Medicine*, *19*(1), 9–18. 10.3892/etm.2019.8213 31897093 10.3892/etm.2019.8213 PMC6923752

[57] Qin, Z., Li, Y., Li, Y., & Liu, G. (2015). Tumor necrosis factor alpha stimulates proliferation of dental pulp stem cells via Akt/Glycogen Synthase Kinase-3β/Cyclin D1 signaling pathway. *Journal of Endodontia*, *41*(7), 1066–1072. 10.1016/j.joen.2015.02.020 10.1016/j.joen.2015.02.020 25843750

[58] Qu, C., Brohlin, M., Kingham, P. J., & Kelk, P. (2020). Evaluation of growth, stemness, and angiogenic properties of dental pulp stem cells cultured in cGMP xeno-/serum-free medium. *Cell and Tissue Research*, *380*(1), 93–105. 10.1007/s00441-019-03160-1 31889209 10.1007/s00441-019-03160-1

[59] Raj, A. T., Kheur, S., Khurshid, Z., Sayed, M. E., Mugri, M. H., Almasri, M. A., Al-Ahmari, M. M., Patil, V. R., Bhandi, S., Testarelli, L., & Patil, S. (2021). The growth factors and cytokines of dental pulp mesenchymal stem cell secretome may potentially aid in oral cancer proliferation. *Molecules*, *26*(18). 10.3390/molecules26185683 10.3390/molecules26185683 PMC8466568 34577154

[60] Roato, I., Baima, G., Orrico, C., Mosca Balma, A., Alotto, D., Romano, F., Ferracini, R., Aimetti, M., & Mussano, F. (2023). Senescent markers expressed by periodontal ligament-derived stem cells (PDLSCs) harvested from patients with periodontitis can be rejuvenated by RG108. *Biomedicines*, *11*(9). 10.3390/biomedicines11092535 10.3390/biomedicines11092535 PMC10526252 37760976

[61] Roederer, M., & Tárnok, A. (2010). OMIPs—Orchestrating multiplexity in polychromatic science. *Cytometry Part A*, *77A*(9), 811–812. 10.1002/cyto.a.20959 10.1002/cyto.a.20959 20722007

[62] Sanz-Serrano, D., Sánchez-de-Diego, C., Mercade, M., & Ventura, F. (2023). Dental Stem Cells SV40, a new cell line developed in vitro from human stem cells of the apical papilla. *International Endodontic Journal*, *56*(4), 502–513. 10.1111/iej.13887 36585930 10.1111/iej.13887

[63] Shekatkar, M. R., Kheur, S. M., Kharat, A. H., Deshpande, S. S., Sanap, A. P., Kheur, M. G., & Bhonde, R. R. (2022). Assessment of angiogenic potential of mesenchymal stem cells derived conditioned medium from various oral sources. *Journal of Clinical and Translational Research*, *8*(4), 323–338. 36090765 PMC9450500

[64] Sonmez Kaplan, S., Sazak Ovecoglu, H., Genc, D., & Akkoc, T. (2023). TNF-α, IL-1B and IL-6 affect the differentiation ability of dental pulp stem cells. *BMC Oral Health*, *23*(1), 555. 10.1186/s12903-023-03288-1 37568110 10.1186/s12903-023-03288-1 PMC10422753

[65] Staniowski, T., Zawadzka-Knefel, A., & Skośkiewicz-Malinowska, K. (2021). Therapeutic potential of dental pulp stem cells according to different transplant types. *Molecules*, *26*(24). 10.3390/molecules26247423 10.3390/molecules26247423 PMC8707085 34946506

[66] Su, Y., Liu, D., Liu, Y., Zhang, C., Wang, J., & Wang, S. (2015). Physiologic levels of endogenous hydrogen sulfide maintain the proliferation and differentiation capacity of periodontal ligament stem cells. *Journal of Periodontology*, *86*(11), 1276–1286. 10.1902/jop.2015.150240 26269939 10.1902/jop.2015.150240

[67] Subhi, H., Husein, A., Mohamad, D., Nik Abdul Ghani, N. R., & Nurul, A. A. (2021). Chitosan-based accelerated portland cement promotes dentinogenic/osteogenic differentiation and mineralization activity of SHED. *Polymers*, *13*(19). 10.3390/polym13193358 10.3390/polym13193358 PMC8512062 34641172

[68] Tanavde, V., Vaz, C., Rao, M. S., Vemuri, M. C., & Pochampally, R. R. (2015). Research using mesenchymal stem/stromal cells: Quality metric towards developing a reference material. *Cytotherapy*, *17*(9), 1169–1177. 10.1016/j.jcyt.2015.07.008 26276001 10.1016/j.jcyt.2015.07.008 PMC4943322

[69] Tomasello, L., Mauceri, R., Coppola, A., Pitrone, M., Pizzo, G., Campisi, G., Pizzolanti, G., & Giordano, C. (2017). Mesenchymal stem cells derived from inflamed dental pulpal and gingival tissue: A potential application for bone formation. *Stem Cell Research & Therapy*, *8*(1), 179. 10.1186/s13287-017-0633-z 28764802 10.1186/s13287-017-0633-z PMC5540218

[70] Trimmel, K., Cvikl, B., Müller, H. D., Nürnberger, S., Gruber, R., Moritz, A., & Agis, H. (2015). L-mimosine increases the production of vascular endothelial growth factor in human tooth slice organ culture model. *International Endodontic Journal*, *48*(3), 252–260. 10.1111/iej.12307 24786562 10.1111/iej.12307

[71] Tsai, C. L., Chuang, P. C., Kuo, H. K., Chen, Y. H., Su, W. H., & Wu, P. C. (2015). Differentiation of stem cells from human exfoliated deciduous teeth toward a phenotype of corneal epithelium in vitro. *Cornea*, *34*(11), 1471–1477. 10.1097/ico.0000000000000532 26165791 10.1097/ICO.0000000000000532

[72] Turrioni, A. P., Oliveira Neto, N. F., Xu, Y., Morse, L., Costa, C. A. S., Battaglino, R., & Hebling, J. (2021). Proliferation rate and expression of stem cells markers during expansion in primary culture of pulp cells. *Brazilian Oral Research*, *35*, e128. 10.1590/1807-3107bor-2021.vol35.0128 34878083 10.1590/1807-3107bor-2021.vol35.0128

[73] Vasandan, A. B., Shankar, S. R., Prasad, P., Sowmya Jahnavi, V., Bhonde, R. R., & Jyothi Prasanna, S. (2014). Functional differences in mesenchymal stromal cells from human dental pulp and periodontal ligament. *Journal of Cellular and Molecular Medicine*, *18*(2), 344–354. 10.1111/jcmm.12192 24393246 10.1111/jcmm.12192 PMC3930420

[74] Wada, N., Gronthos, S., & Bartold, P. M. (2013). Immunomodulatory effects of stem cells. *Periodontol 2000l*, *63*(1), 198–216. 10.1111/prd.12024 10.1111/prd.12024 23931061

[75] Wang, M., Xie, J., Wang, C., Zhong, D., Xie, L., & Fang, H. (2020). Immunomodulatory properties of stem cells in periodontitis: Current status and future prospective. *Stem Cells International*, *2020*, 9836518. 10.1155/2020/9836518 32724318 10.1155/2020/9836518 PMC7366217

[76] Wang, X., Li, F., Wu, S., Xing, W., Fu, J., Wang, R., & He, Y. (2024). Research progress on optimization of in vitro isolation, cultivation and preservation methods of dental pulp stem cells for clinical application [Review]. *Frontiers in Bioengineering and Biotechnology*, *12*. 10.3389/fbioe.2024.1305614 10.3389/fbioe.2024.1305614 PMC11021638 38633667

[77] Wang, X. T., Rao, N. Q., Fang, T. J., Zhao, Y. M., & Ge, L. H. (2018). Comparison of the properties of CD146 positive and CD146 negative subpopulations of stem cells from human exfoliated deciduous teeth. *Beijing Da Xue Xue Bao Yi Xue Ban*, *50*(2), 284–292. 29643528

[78] Widbiller, M., Eidt, A., Wölflick, M., Lindner, S. R., Schweikl, H., Hiller, K. A., Buchalla, W., & Galler, K. M. (2018). Interactive effects of LPS and dentine matrix proteins on human dental pulp stem cells. *International Endodontic Journal*, *51*(8), 877–888. 10.1111/iej.12897 29377169 10.1111/iej.12897

[79] Xiang, X., Hu, Y., Song, Z., & Wang, C. (2023). Knockdown of TRPM2 promotes osteogenic differentiation of human periodontal ligament stem cells by modulating NF-κB /NLRP3 pathway. *Tissue and Cell*, *84*, 102184. 10.1016/j.tice.2023.102184 37541115 10.1016/j.tice.2023.102184

[80] Xin, T., Li, Q., Bai, R., Zhang, T., Zhou, Y., Zhang, Y., Han, B., & Yang, R. (2021). A novel mutation of SATB2 inhibits odontogenesis of human dental pulp stem cells through Wnt/β-catenin signaling pathway. *Stem Cell Research & Therapy*, *12*(1), 595. 10.1186/s13287-021-02660-8 34863303 10.1186/s13287-021-02660-8 PMC8642962

[81] Xu, J. G., Zhu, S. Y., Heng, B. C., Dissanayaka, W. L., & Zhang, C. F. (2017). TGF-β1-induced differentiation of SHED into functional smooth muscle cells. *Stem Cell Research & Therapy*, *8*(1), 10. 10.1186/s13287-016-0459-0 28114966 10.1186/s13287-016-0459-0 PMC5260045

[82] Yamada, Y., Nakamura, S., Ito, K., Sugito, T., Yoshimi, R., Nagasaka, T., & Ueda, M. (2010). A feasibility of useful cell-based therapy by bone regeneration with deciduous tooth stem cells, dental pulp stem cells, or bone-marrow-derived mesenchymal stem cells for clinical study using tissue engineering technology. *Tissue Engineering Part A*, *16*(6), 1891–1900. 10.1089/ten.TEA.2009.0732 20067397 10.1089/ten.TEA.2009.0732

[83] Yamada, Y., Nakamura-Yamada, S., Umemura-Kubota, E., & Baba, S. (2019). Diagnostic Cytokines and Comparative Analysis Secreted from Exfoliated Deciduous Teeth, Dental Pulp, and Bone Marrow Derived Mesenchymal Stem Cells for Functional Cell-Based Therapy. *International Journal of Molecular Sciences*, *20*(23), 5900. 10.3390/ijms20235900 31771293 10.3390/ijms20235900 PMC6928984

[84] Yamaza, T., Kentaro, A., Chen, C., Liu, Y., Shi, Y., Gronthos, S., Wang, S., & Shi, S. (2010). Immunomodulatory properties of stem cells from human exfoliated deciduous teeth. *Stem Cell Research & Therapy*, *1*(1), 5. 10.1186/scrt5 20504286 10.1186/scrt5 PMC2873699

[85] Yang, S., Guo, L., Su, Y., Wen, J., Du, J., Li, X., Liu, Y., Feng, J., Xie, Y., Bai, Y., Wang, H., & Liu, Y. (2018). Nitric oxide balances osteoblast and adipocyte lineage differentiation via the JNK/MAPK signaling pathway in periodontal ligament stem cells. *Stem Cell Research & Therapy*, *9*(1), 118. 10.1186/s13287-018-0869-2 29716662 10.1186/s13287-018-0869-2 PMC5930947

[86] Ye, L., Yu, Z., He, L., Yuan, J., Zhang, X., Li, L., Huang, X., Ma, Y., & Zhang, L. (2024). KAT2A-mediated succinylation modification of notch1 promotes the proliferation and differentiation of dental pulp stem cells by activating notch pathway. *BMC Oral Health*, *24*(1), 407. 10.1186/s12903-024-03951-1 38556862 10.1186/s12903-024-03951-1 PMC10981825

[87] Yi, B., Ding, T., Jiang, S., Gong, T., Chopra, H., Sha, O., Dissanayaka, W. L., Ge, S., & Zhang, C. (2021). Conversion of stem cells from apical papilla into endothelial cells by small molecules and growth factors. *Stem Cell Research & Therapy*, *12*(1), 266. 10.1186/s13287-021-02350-5 33941255 10.1186/s13287-021-02350-5 PMC8091697

[88] Yu, S., Liu, X. M., Liu, Y., Tang, L., Lei, S., Geng, C., Yuan, Z., & Chen, X. (2024). Inflammatory microenvironment of moderate pulpitis enhances the osteo-/odontogenic potential of dental pulp stem cells by autophagy. *International Endodontic Journal*. 10.1111/iej.14108 10.1111/iej.14108 39031653

[89] Zhang, W., Zhang, X., Ling, J., Wei, X., & Jian, Y. (2016). Osteo-/odontogenic differentiation of BMP2 and VEGF gene-co-transfected human stem cells from apical papilla. *Molecular Medicine Reports*, *13*(5), 3747–3754. 10.3892/mmr.2016.4993 26986020 10.3892/mmr.2016.4993 PMC4838134

[90] Zheng, J., Kong, Y., Hu, X., Li, Z., Li, Y., Zhong, Y., Wei, X., & Ling, J. (2020). MicroRNA-enriched small extracellular vesicles possess odonto-immunomodulatory properties for modulating the immune response of macrophages and promoting odontogenesis. *Stem Cell Research & Therapy*, *11*(1), 517. 10.1186/s13287-020-02039-1 33256846 10.1186/s13287-020-02039-1 PMC7708107

